# Selective isomerization of 2,3-disubstituted butanediacetal esters and thioesters using lithium and sodium enolates: effects of bases and additives on stereoselectivity

**DOI:** 10.3389/fchem.2026.1750600

**Published:** 2026-02-04

**Authors:** Adam Drop, Magdalena Grzegolec, Bożena Frąckowiak-Wojtasek

**Affiliations:** Faculty of Chemistry, Opole University, Opole, Poland

**Keywords:** butanediacetals (BDAs), enolate ions, hexamethylphosphoramide (HMPA), isomerization, lithium chloride, lithium diisopropylamide (LDA), sodium isopropyl (trimethylsilyl)amide (NaPTA)

## Abstract

The study concerned the stereoselective isomerisation of S2,S3-diethyl (*2R,3R,5R,6R*)-5,6-dimethoxy-5,6-dimethyl-1,4-dioxane-2,3-dicarbotioate to the isomer S2, S3-diethyl (*2R,3S,5R,6R*)-5,6-dimethoxy-5,6-dimethyl-1,4-dioxane-2,3-dicarbotioate via stable lithium and sodium enolates. For this purpose, enolate anions were generated using lithium diisopropylamide (LDA) and sodium isopropyl(trimethylsilyl)amide (NaPTA), using methanol as a proton source. Optimal stereoselectivity was achieved using 2.2 equivalents of LDA or NaPTA, yielding isomer ratios of 1:6.3 and 1:6.1, respectively, and good yields of 68% and 79%. The selectivity of the reaction was further increased in the presence of 10% (v/v) HMPA or six equivalents of LiCl in systems with LDA, indicating the important role of metal complexation. A symmetrical bis(silyl enol ether) with a Z,Z configuration of double bonds was isolated from the enolate formed during the reaction, confirming the ordered geometry of the system. The observed stereoselectivity is explained by the non-equivalence of the two possible enolates and the chelation-controlled desymmetrisation of the dienolate. Both NaPTA and LDA enabled selective isomerisation without additives, with NaPTA proving particularly effective for related butanediacetal derivatives containing thioesters, providing >90% cis isomer yield for the mixed ester–thioester substrate. These results show that NaPTA is an efficient and highly stereoselective base for the isomerisation of thioester-containing butanediacetals, expanding the possibilities of stereocontrolled enolate chemistry.

## 1 Introduction

**SCHEME 1:**
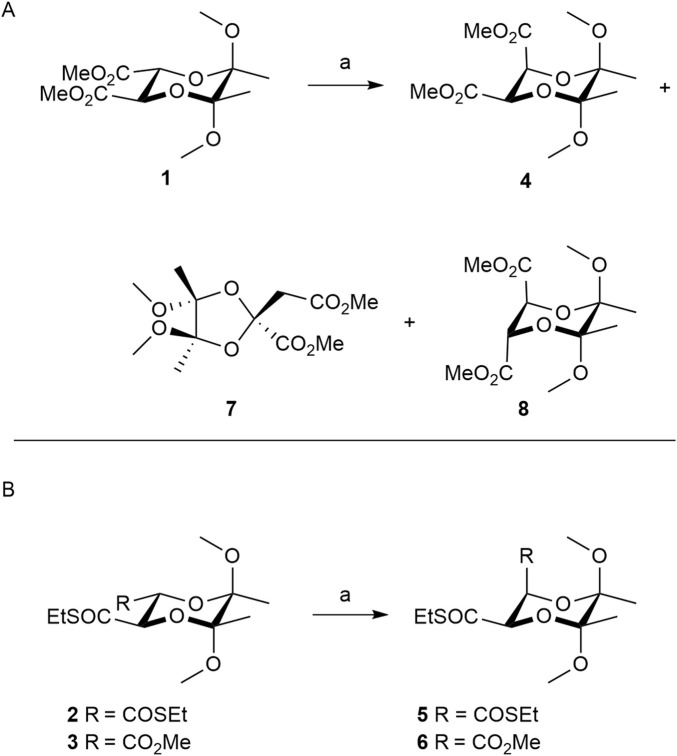
**(A)** Isomerization of diester BDA derivative **1**. **(B)** Isomerization of thioester BDA derivatives **2** and **3**. Reagents: a) 1, 2.2 eq. of LDA, −78 °C, 2. MeOH.

Butanediacetals (BDAs) represent a significant class of compounds employed in the field of organic synthesis (Lence et al., 2008; Ley et al., 2001; Ley and Polara, 2007). These compounds function as building blocks, capable of initiating stereoselective reactions while concurrently protecting 1,2-diols, or alternatively, providing a selective blocking group (Barros et al., 2004; Brittain et al., 2005; Buckler et al., 2017a; Buckler et al., 2017b; Burke et al., 2006; Drop et al., 2020; Johnson et al., 2021; Kiyota et al., 2002; Kleinbeck et al., 2012; Leyva et al., 2008; Mahapatra and Carter, 2009; Miranda et al., 2015; Nattrass et al., 2005; Pohmakotr et al., 2008; Shing et al., 2008; Shing et al., 2009; Wheatley et al., 2021; Woodhall et al., 2005). Some of them are obtained easily in large quantities from (*R*,*R*)-and (*S*,*S*)-dimethyl tartrate such as ester or thioester *trans*-2,3-disubstituted analogus **1**-**3** (Scheme 1) (Dixon et al., 2001; Maycock and Ventura, 2012).

*cis*-2,3-Disubstituted derivatives of butanediacetals **4**-**6**, which are products of the isomerisations of *trans*-BDAs **1**-**3** according to Scheme 1, are of significant importance in the synthesis of natural products and ligands used in organocatalysis (Burke et al., 2006; Dixon et al., 2001; Kiyota et al., 2002; Dixon et al., 2002). Consequently, isomerization reactions of *trans* derivatives **1**-**3** to *cis* isomers **4**-**6** are developed (Barros et al., 1999; Dixon et al., 2001; Drop et al., 2017; Maycock and Ventura, 2012). These isomerizations are carried out via the enolate ions of compounds **1**-**3** by treatment with a base: lithium diisopropylamide (LDA), and then adding methanol as a proton source. However, attempts to isomerize the diester derivative **1** in this way gave a mixture of products with compound **7** as the major component (Scheme 1A) (Barros et al., 1999). In another method treatment of the dienolate of **1** with iodine gave the blocked maleate derivative as the product of the double elimination reaction, which can be catalytically reduced with hydrogen at high pressure (∼80 bar) to the diastereoisomer **4**, in which one ester group is in the equatorial position and the other one is in the axial position (Dixon et al., 2001). To avoid the high pressure reduction compound **1** may be replaced with compound **2**, in which the methyl ester groups are replaced by ethyl thioesters (Scheme 1B) (Maycock and Ventura, 2012). The dienolate obtained from the dithioester **2** treated with methanol give products different than the diester derivative **1**. In this case a mixture of diastereoisomers **5** and **2** was synthesized in a ratio 1.6:1, without any rearrangement products. The most selective isomerization is the one using a butanediacetal derivative **3** having one ester group and one thioester group (Scheme 1B) (Drop et al., 2017). This reaction led to the formation of product **6** selectively in a high yield of 94%. However, the synthesis of substrate **3** from diester **1** occurs with a maximum yield of 63%. In all the reactions described above, enolate ions are obtained using only the strongest base LDA.

Sodium isopropyl (trimethylsilyl)amide (NaPTA) is a base that was introduced in 2022 by Collum’s group (Ma et al., 2022). The structural formula of this base consists of isopropylamine, a structural fragment of the strongest base (LDA), as well as a trimethylsilyl group, which is present in the weakest bases, lithium, potassium, or sodium hexamethyldisilazanes. It has been demonstrated that NaPTA exhibits higher reactivity in comparison to LDA in the context of metalation reactions involving arenes, epoxides, ketones, hydrazones, alkenes, and alkyl halides. Alkylation reactions of Oppolzer enolates, where NaPTA is used, proceed in the presence of HMPA (Lui and Collum, 2023). Aminolysis of methyl benzoate in the presence of NaPTA gives benzamides (You et al., 2024). NaPTA is kinetically more reactive and more stable than LDA, and its solubility in THF is good. LDA is a base that has had a special place in organometallic chemistry since 1950 when it was first synthesised (Braun, 2015; Collum et al., 2007; Hamell and Levine, 1950; Wu and Huang, 2006). However, recent research is moving towards sodium organic chemistry which is related to the greater occurrence of sodium than lithium in nature and the good reactivity of organosodium compounds (Anderson et al., 2024a; Anderson et al., 2024b; De et al., 2021; Gentner and Mulvey, 2021; Mulvey and Robertson, 2013; Neil et al., 2025; Tong et al., 2022; Woltornist et al., 2020; You et al., 2024).

To date, the isomerization of butanediacetal derivatives **1**, **2**, and **3** has been carried out exclusively using the strongest base, LDA. The isomerization of diester derivative **1** yields a mixture of compounds, and the same reaction with compound **3**, despite its complete selectivity, has an earlier stage with average yield. Therefore, we undertook to optimize the isomerization reaction of the dithioester analogue BDA **2** using LDA and NaPTA. In light of the previously outlined data concerning the novel NaPTA base, a decision was made to undertake inversion reactions of derivatives **1**, **2**, and **3** utilizing the NaPTA base.

## 2 Materials and methods

All non-aqueous reactions were carried out under an atmosphere of argon and in oven-dried glassware. Chemicals were purchased from Merck, Fluorochem or Avantor Performance Materials S.A. Poland. Chemicals and solvents were of analytica grade. Tetrahydrofuran (THF) was distilled from sodium/benzophenone under argon. Diisopropylamine was dried over KOH for 24 h and then distilled under argon. ^1^H NMR, ^13^C NMR spectra were recorded on a Bruker Avance 400 MHz spectrometer. ^1^H NMR spectra were obtained at 400 MHz in CDCl_3_ with chemical shift (δ) values in ppm downfield from tetramethylsilane, coupling constants (*J*) were expressed in Hz. ^13^C NMR spectra were obtained at 100.62 MHz in CDCl_3_. Analytical TLC was done on precoated aluminum plates (Merck silica gel 60 F254 or Supelco silica gel) and visualized by UV fluorescence and aqueous solution of potassium permanganate. Preparative column chromatography was done on silica gel (60–120 mesh, Merck). Optical rotations were measured with a Jasco P-2000 polarimeter. Melting points (uncorrected) were determined on a Boetius apparatus. IR spectra were recorded as thin films between potassium bromide plates or as KBr pastilles in a Nicolet 6700 spectrometer. High resolution mass spectra (HRMS) were obtained on a Bruker Daltonik micrOTOF-Q II mass spectrometer equipped with an ESI source in the positive ion mode calibrated with sodium formate clusters. (*2R*,*3R*,*5R*,*6R*)-5,6-Dimethoxy-5,6-dimethyl-1,4-dioxane-2,3-dimethyl dicarboxylate **1** was obtained from dimethyl L-tartrate according to published procedure (Dixon et al., 2001). S2,S3-Diethyl (*2R*,*3R*,*5R*,*6R*)-5,6-dimethoxy-5,6-dimethyl-1,4-dioxane-2,3-dicarbothioate **2** was synthesized from (+)-dimethyl L-tartrate according to published procedures (Maycock and Ventura, 2012). Methyl (2*R*,3*R*,5*R*,6*R*)-3-ethylsulfanylcarbonyl-5,6-dimethoxy-5,6-dimethyl-1,4-dioxane-2-carboxylate **3** was synthesized from (+)-dimethyl L-tartrate according to published procedure (Drop et al., 2017). Sodium isopropyl (trimethylsilyl)amide (NaPTA) was obtained according to published procedure (Ma et al., 2022). Isopropyl (trimethylsilyl)amine was synthesized from isopropylamine and trimetylsilyl chloride (Gaul et al., 2000). Concentration of sodium isopropyl (trimethylsilyl)amide (NaPTA) was determined by titration with diphenylacetic acid (Kofron and Baclawski, 1976).

Isomerization of S2,S3-diethyl (*2R*,*3R*,*5R*,*6R*)-5,6-dimethoxy-5,6-dimethyl-1,4-dioxane-2,3-dicarbothioate 2

### 2.1 General procedure for reactions with LDA

*n*-Butyllithium (0.50 mL, 1.26 mmol) was added dropwise to a solution of diisopropylamine (0.19 mL, 1.38 mmol) in anhydrous THF (1.06 mL) at −30 °C. The solution was stirred for 30 min and then cooled down to −78 °C. S2,S3-Diethyl (*2R*,*3R*,*5R*,*6R*)-5,6-dimethoxy-5,6-dimethyl-1,4-dioxane-2,3-dicarbothioate **2** (200 mg, 0.57 mmol) in THF (1 mL) was added to the mixture, which was then stirred for 30 min at −78 °C. Next, anhydrous methanol (0.57 mL) was added dropwise, the stirring was continued for 15 min at −78 °C, and then the reaction was stopped by addition of saturated ammonium chloride solution at −78 °C and extracted three times with dichloromethane. The combined organic phases were washed with brine and distilled water, dried over sodium sulfate. After evaporation of the solvent the mixture of products was separated by column chromatography (eluent 20% ethyl acetate in petroleum ether) giving product **5** in a mixture with **2** 151 mg (0.43 mmol) with yield 75% for the reaction with 2.2 eq of LDA. An analogous procedure was used for reactions with other LDA equivalents and other alcohols.

Compound **2**:

^1^H NMR (CDCl_3_): δ = 4.47 (1H, s), 3.31 (3H, s), 2.91 (2H, qd, *J* = 7.4 Hz, *J* = 1.0 Hz), 1.36 (3H, s), 1.27 (3H, t, *J* = 7.6 Hz).

^13^C NMR (CDCl_3_): δ = 196.03, 99.68, 74.36, 48.63, 23.03, 17.48, 14.36.

HMRS (+ESI): *m/z* calcd for C_14_H_24_NaO_6_S_2_ [M+Na]^+^ 375,0907; found: 375,0939.

Compound **5**:

^1^H NMR (CDCl_3_): δ = 4.71 (1H, d, *J* = 4.2 Hz), 4.62 (1H, d, *J* = 4.2 Hz), 3.32 (3H, s), 3.23 (3H, s), 2.93-2.81 (4H, m), 1.41 (3H, s), 1.35 (3H, s), 1.27 (3H, t, *J* = 7.4 Hz), 1.25 (3H, t, *J* = 7.4 Hz).

HMRS (+ESI): *m/z* calcd for C_14_H_24_NaO_6_S_2_ [M+Na]^+^ 375,0907; found: 375,0918.

### 2.2 Reactions in the presence of HMPA

After the addition of compound **2** (Procedure 2.1), a 10% solution of HMPA in anhydrous methanol (0.57 mL) was added dropwise, and the mixture was stirred for 15 min at −78 °C. The workup was carried out as described in Procedure 2.1. After evaporation of the solvent, the product mixture was separated by column chromatography (eluent: 20% ethyl acetate in petroleum ether), affording product **5** as a mixture with compound **2** (151 mg, 0.43 mmol, 75% yield).

### 2.3 Reactions with lithium chloride

Lithium chloride (148 mg, 3.42 mmol) was added into the flask prior to the addition of diisopropylamine and *n*-butyllithium (procedure 2.1). The reaction mixture was processed as described in procedure 2.1. After evaporation of the solvent the mixture of products were resolved by column chromatography (eluent 20% ethyl acetate in petroleum ether) giving product **5** in a mixture with **2** 140 mg (0.40 mmol) with yield 70%.

### 2.4 General procedure for reactions with NaPTA

A solution of sodium isopropyl (trimethylsilyl)amide (0.84 mL, 1.5 M in THF, 1.26 mmol) was added dropwise to a solution of S2,S3-diethyl (*2R*,*3R*,*5R*,*6R*)-5,6-dimethoxy-5,6-dimethyl-1,4-dioxane-2,3-dicarbothioate **2** (200 mg, 0.57 mmol) in anhydrous THF (2 mL) at −78 °C. The reaction mixture was stirred for 30 min at −78 °C. Next, anhydrous methanol (0.57 mL) was added dropwise, the stirring was continued for 30 min at −78 °C, and then the reaction was stopped by addition of saturated ammonium chloride solution at −78 °C and extracted three times with dichloromethane. The combined organic phases were washed with brine and distillated water, dried over sodium sulfate. After evaporation of the solvent the mixture of products were resolved by column chromatography (eluent 20% ethyl acetate in petroleum ether) giving product **5** in a mixture with **2** 158 mg (0.45 mmol) with yield 79%.

### 2.5 Reactions NaPTA in the presence of HMPA or TMEDA

After addition of compound **2** (procedure 2.4) 10% HMPA or 10% TMEDA in anhydrous methanol (0.57 mL) was added dropwise and the mixture was stirred for 15 min at −78 °C. The workup was the same as in procedure 2.4. After evaporation of the solvent the mixture of products were resolved by column chromatography (eluent 20% ethyl acetate in petroleum ether) giving product **5** in a mixture with **2** with yield 75% for HMPA and 66% for TMEDA.

### 2.6 Reactions NaPTA with lithium chloride

Lithium chloride (148 mg, 3.42 mmol) was added into the flask prior to the addition of NaPTA (procedure 2.4). The reaction mixture was processed as described in procedure 2.4. After evaporation of the solvent the mixture of products were resolved by column chromatography (eluent 20% ethyl acetate in petroleum ether) giving product **5** in a mixture with **2** 175 mg (0.50 mmol) with yield 87%.

### 2.7 Preparation of silyl enol ethers 10

After addition of compound **2** to LDA (as in procedure 1.1) and stirring for 30 min at −78 °C, HMPA (0.322 mL) was added follow by addition of trimethylsilyl chloride (0.36 mL, 2.85 mmol) was added dropwise, the mixture was stirred for 15 min at −78 °C, then the reaction was stopped and worked up in the same way as in procedure 2.1 without purification on chromatographic column.

Silyl enol ether **10**: IR (film): 1,681, 1,622, 1,166, 1,142 cm^-1^; ^1^H NMR (CDCl_3_): δ = 3.37 (3H, s), 2.84-2.68 (2H, m), 1.37 (3H, s), 1.25 (3H, t, *J* = 7.4 Hz), 0.20 (9H, s). ^13^C NMR (CDCl_3_): δ = 135.46, 129.34, 101.11, 49.96, 24.83, 17.66, 15.03, 0.24. HMRS (+ESI): *m/z* calcd for C_20_H_40_NaO_6_S_2_Si_2_ [M+Na]^+^ 519.1697; found: 519.1730.

### 2.8 Isomerization of (*2R*,*3R*,*5R*,*6R*)-5,6-dimethoxy-5,6-dimethyl-1,4-dioxane-2,3-dimethyl dicarboxylate 1 with NaPTA

A solution of sodium isopropyl (trimethylsilyl)amide (1.5 mL, 1.5 M in THF, 2.2 mmol) was added dropwise to a solution of (2*R*,3*R*,5*R*,6*R*)-5,6-dimethoxy-5,6-dimethyl-1,4-dioxane-2,3-dimethyl dicarboxylate **1** (0.29 g, 1.0 mmol) in anhydrous THF (4 mL) at −78 °C and the reaction mixture was then stirred for 30 min at −78 °C. Next, anhydrous methanol (1.0 mL) was added dropwise, the stirring was continued for 30 min at −78 °C, and then the reaction was stopped by addition of saturated ammonium chloride solution at −78 °C and extracted three times with dichloromethane. The combined organic phases were washed with brine and distillated water, dried over sodium sulfate. After evaporation of the solvent the mixture of products were resolved by column chromatography (eluent with gradient: 10%–40% ethyl acetate in petroleum ether) giving products: **4** (26 mg), **7** in mixture of **1** (173 mg), and **8** (69 mg). NMR spectra of the substrate **1**, crude reaction mixture and products **4**, **7**, and **12** are presented respectively in:

Compound **1**

^1^H NMR (CDCl_3_): 4.52 (s, 2H), 3.75 (s, 6H), 3.31 (s, 6H), 1.35 (s, 6H);

^13^C NMR (CDCl_3_): δ = 168.56, 99.31, 68.86, 52.66, 48.58, 17.44;

HRMS (+ESI): *m/z* calcd for C_12_H_20_NaO_8_ [MNa^+^] 315.1050, found: 315.1041.

Compound **4**

^1^H NMR (CDCl_3_): δ = 4.69 (d, *J* = 4 Hz, 1H), 4.50 (d, *J* = 4 Hz, 1H), 3.77 (s, 3H), 3.75 (s, 3H), 3.29 (s, 3H), 3.20 (s, 3H), 1.42 (s, 3H), 1.39 (s, 3H);

^13^C NMR (CDCl_3_): δ = 169.92, 169.01, 100.66, 99.47, 69.27, 66.23, 52.43, 52.20, 50.39, 48.69, 18.05, 17.55

HRMS (+ESI): *m/z* calcd for C_12_H_20_NaO_8_ [MNa^+^] 315.1050, found: 315.1062.

Compound **7**

^1^H NMR (CDCl_3_): δ = 3.80 (s, 3H), 3.67 (s, 3H), 3.32 (s, 3H), 3.25 (s, 3H), 3.08 (d, *J* = 15 Hz, 1H), 2.90 (d, *J* = 15 Hz, 1H), 1.38 (s, 3H), 1.33 (s, 3H);

^13^C NMR (CDCl_3_): δ = 169.40, 168.93, 109.25, 108.74, 103.62, 52.82, 52.09, 49.35, 49.14, 43.89, 15.36, 15.09;

HRMS (+ESI): *m/z* calcd for C_12_H_20_NaO_8_ [MNa^+^] 315.1050, found: 315.1043.

Compound **8**

^1^H NMR (CDCl_3_): δ = 4.82 (s, 3H), 3.76 (s, 3H), 3.38 (s, 3H),1.38 (s, 3H);

^13^C NMR (CDCl_3_): δ = 170.00, 100.44, 70.14, 52.54, 49.43, 18.26;

HRMS (+ESI): *m/z* calcd for C_12_H_20_NaO_8_ [MNa^+^] 315.1050, found: 315.1064.

### 2.9 Isomerization of methyl (2*R*,3*R*,5*R*,6*R*)-3-ethylsulfanylcarbonyl-5,6-dimethoxy-5,6-dimethyl-1,4-dioxane-2-carboxylate 3 with NaPTA

A solution of sodium isopropyl (trimethylsilyl)amide (0.84 mL, 1.5 M in THF, 1.25 mmol) was added dropwise to a solution of methyl (2*R*,3*R*,5*R*,6*R*)-3-ethylsulfanylcarbonyl-5,6-dimethoxy-5,6-dimethyl-1,4-dioxane-2-carboxylate **3** (0.16 g, 0.5 mmol) in anhydrous THF (2 mL) at −78 °C and the reaction mixture was then stirred for 30 min at −78 °C. Next, anhydrous methanol (1.0 mL) was added dropwise, the stirring was continued for 30 min at −78 °C, and then the reaction was stopped by addition of saturated ammonium chloride solution at −78 °C and extracted three times with dichloromethane. The combined organic phases were washed with brine and distillated water, dried over sodium sulfate. After evaporation of the solvent the mixture of products were resolved by column chromatography (eluent 15% ethyl acetate in petroleum ether) giving product: **6** 144 mg (0.45 mmol), yield 90%. NMR spectra of the substrate **3**, crude reaction mixture and products **6**, are presented respectively in:

Compound **3**

^1^H NMR (CDCl_3_): 4.58 (d, *J* = 9.84 Hz, 1H), 4.36 (d, *J* = 9.84 Hz, 1H), 3.78 (s, 3H), 3.33 (s, 3H), 3.29 (s, 3H), 2.87 (q, *J* = 7.44 Hz, 2H), 1.38 (s, 3H), 1.33 (s, 3H), 1.25 (t, *J* = 7.44 Hz, 3H);

^13^C NMR (CDCl_3_): δ = ; 197.50, 168.30, 100.00, 99.18, 74.21, 69.63, 52.70, 48.68, 48.56, 22.64, 17.53, 17.42, 14.37;

HRMS (+ESI): *m/z* calcd for C_13_H_22_NaO_7_S [MNa^+^] 345.0978, found: 345.0974.

Compound **6**

^1^H NMR (CDCl_3_): 4.62 (d, *J* = 3.88 Hz, 1H), 4.54 (d, *J* = 3.84 Hz, 1H), 3.73 (s, 3H), 3.31 (s, 3H), 3.20 (s, 3H), 2.98-2.80 (m, 2H), 1.41 (s, 3H), 1.33 (s, 3H), 1.30 (t, *J* = 7.52 Hz, 3H);

^13^C NMR (CDCl_3_): δ = 199.02, 169.45, 101.28, 99.86, 72.44, 69.87, 52.02, 50.20, 48.68, 22.13, 18.03, 17.61, 14.45;

HRMS (+ESI): *m/z* calcd for C_13_H_22_NaO_7_S [MNa^+^] 345.0978, found: 345.0982.

## 3 Results and discussion

In the optimization of the isomerization reaction, the initial focus was on the optimization of the isomerization of dithioester **2** to its *cis*-diastereomer **5**. A systematic examination was conducted to determinate the influence of the base, stoichiometry, reaction time, temperature, proton source, and additives on reaction selectivity and yield (Table 1). First, the influence of LDA equivalents to dithioester 2 on reaction selectivity was verified. When 2.2 eq. of LDA were used and the generation of the dienolate was carried out for 30 min at −78 °C followed by the addition of methanol a mixture of diastereoisomers **5** and **2** was obtained in a ratio 3:1, which was a significant improvement over the 1.6:1 value reported previously in the literature (Table 1, entry 1). In the ^1^H NMR spectrum of the crude reaction mixture a small amount of the double deprotonation product **9** (4%) was also identified by comparison of the signals with the data from the literature (Maycock and Ventura, 2012). It was observed that the stoichiometry of LDA was critical; decreasing the amount to 1.1 eq. drastically lowered the selectivity to 0.7:1 (Table 1, entry 2), while increasing it to 3.3 eq. offered a slight improvement (Table 1, entry 3). Low selectivity in the reaction with 1.1 eq. of LDA indicating that substrate **2** was not selectively converted into a monoenolate. Additionally, the formation of a dienolate was observed. Therefore, even if the approach of the proton from the equatorial position of BDA was favorable substrate **2** was still not full converted into an enolate in the reaction. It therefore became obvious that larger amount of LDA promotes the formation of the desired isomer and the formation of a dienolate is essential for achieving high *cis*-selectivity. Subsequently, the reaction parameters were examined. As demonstrated in Table 1, entries 4 and 5, methanol was identified as the optimal proton source in comparison to ethanol and t-butanol. Furthermore, the temporal effects of LDA on the isomer ratios of **5** and **2** were examined (Table 1, entries 1, 6, 7, and 8). Upon the immediate addition of methanol following the application of LDA, the ^1^H NMR spectrum revealed the presence of the *trans* isomer **2** exclusively (Table 1, entry 6). Subsequent to the initial 15-min addition of methanol, a mixture of isomers **5** and **2** was obtained in a 6.3:1 ratio. However, the dimethyl ester **1** was also formed in a 15% yield (Table 1, entry 7). The extension of the dienolate formation to 2 h led to a decrease in the ratio of isomers **5** and **2** to 2.6:1and increased the amount of the β-elimination product to 16% (see Table 1, entry 8). Conversely, changes in the temperature of the reaction did not yield the β-elimination product **9**; instead, they influenced the ratio of isomers **5** and **2** (Table 1, entries 9 and 10). As demonstrated in Table 1, entry 9, the substrate **2** exhibited low conversion when the temperature was reduced to −100 °C. The *trans* dimethyl ester **1** was obtained as the predominant component when methanol was added at 0 °C. However, the isomerization process was found to be highly selective, resulting in the formation of the butanediacetal derivatives **5** and **2** in the ratio 15:1 (Table 1, entry 10). The addition of 10% HMPA in methanol at −78 °C resulted in increased selectivity compared to the reaction without HMPA addition (Table 1, entries 3 and 11). Further improvement was achieved with LiCl (6 equiv.), giving a ratio of 5.8:1 (Table 1, entry 12). Subsequently, NaPTA was utilized instead of LDA during the isomerization of BDA **2** to **5**. In light of the outcomes derived from LDA, the decision was made to employ 2.2 eq. of NaPTA for a duration of 15 min, with methanol serving as the proton source. This condition resulted in a ratio of 6.0:1 for the products **5**:**2**, without the addition of any additives and with no detectable transesterification (Table 1, entry 13). This result is analogous to the optimal outcome observed with the LDA/LiCl system, yet it offers more streamlined reaction conditions. Notably, transesterification leading to methyl diester **1** did not occur. In contrast to the LDA system, the addition of coordinating agents (HMPA, TMEDA) or LiCl had a detrimental effect on the selectivity when using NaPTA (Table 1, entries 14–16). This observation indicates a marked difference in the nature of the sodium dienolate intermediate compared to its lithium counterpart. The isomerization reaction, conducted within the temperature range of −78 °C to 0 °C, resulted in transesterification, leading to the formation of a mixture of dimethyl diesters **1** and **4** in a ratio of 1:0.3 (Table 1, entry 17).

**TABLE 1:** Optimization of the isomerization of dithioester **2** to **5**.

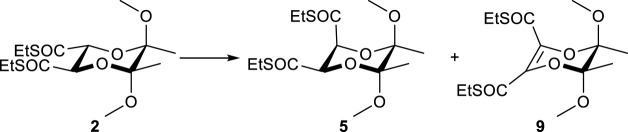					

Entry	Base (eq.)	Time after addition of base (min.)	Alcohol/additives	Temperature (°C)	Ratio of 2:5^a^ (yield of mixture 2, 5%)^b^
1	LDA (2.2)	30	MeOH	−78	1:3 (75)
2	LDA (1.1)	30	MeOH	−78	1:0.7 (76)
3	LDA (3.3)	30	MeOH	−78	1:3.8 (81)
4	LDA (2.2)	30	*t*-BuOH	−78	1:2.3 (80)
5	LDA (2.2)	30	EtOH	−78	1:1.5 (90)
6	LDA (2.2)	0	MeOH	−78	1:0 (-)
7	LDA (2.2)	15	MeOH	−78	1:6.3 (68)
8	LDA (2.2)	120	MeOH	−78	1:2.6 (74)
9	LDA (2.2)	30	MeOH	−100	9:1 (-)
10	LDA (2.2)	30	MeOH	From −78 to 0	1:15 (24)
11	LDA (2.2)	30	MeOH, 10% HMPA	−78	1:5.1 (75)
12	LDA (2.2)	30	MeOH, 6 eq. LiCl	−78	1:5.8 (70)
13	NaPTA (2.2)	15	MeOH	−78	1:6.0 (79)
14	NaPTA (2.2)	30	MeOH, 10% HMPA	−78	1:2.8 (75)
15	NaPTA (2.2)	30	MeOH, 10% TMEDA	−78	1:0.9 (66)
16	NaPTA (2.2)	30	MeOH, 6 eq. LiCl	−78	1:0.8 (87)
17	NaPTA (2.2)	30	MeOH	From −78 to 0	-

^a^ Based on the ^1^H NMR, spectra of the crude products.

^b^ After column chromatography of the mixture of **2** and **5**. The mixture of isomers **2** and **5** cannot be separated on a chromatographic column.

**FIGURE 1:**
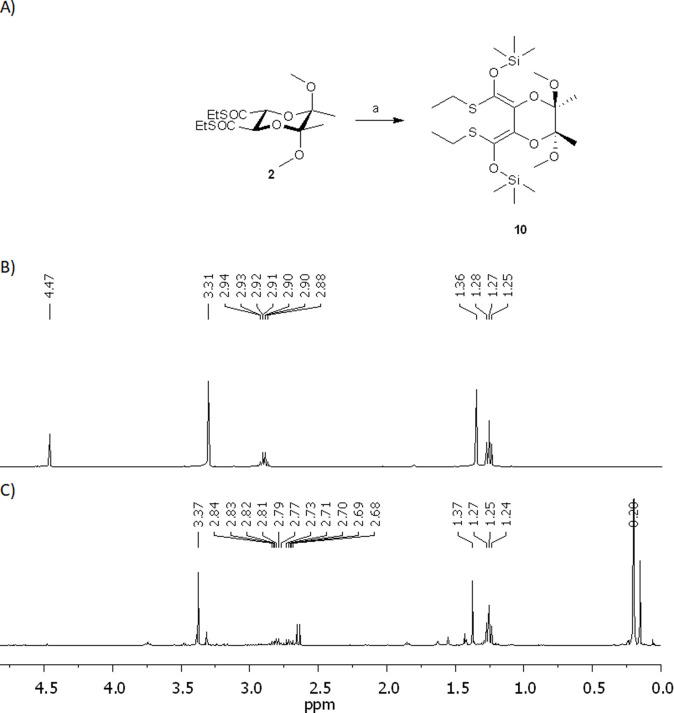
**(A)** Trapping reaction. (a) 2.2 eq. of LDA, - 78 °C, 5 eq. Me_3_SiCl. **(B)** Fragments of ^1^H NMR spectra of the substrate **2**. **(C)** A crude mixture of silyl enol ethers with main product **10** obtained after isomerization of **2** with HMPA.

**FIGURE 2:**
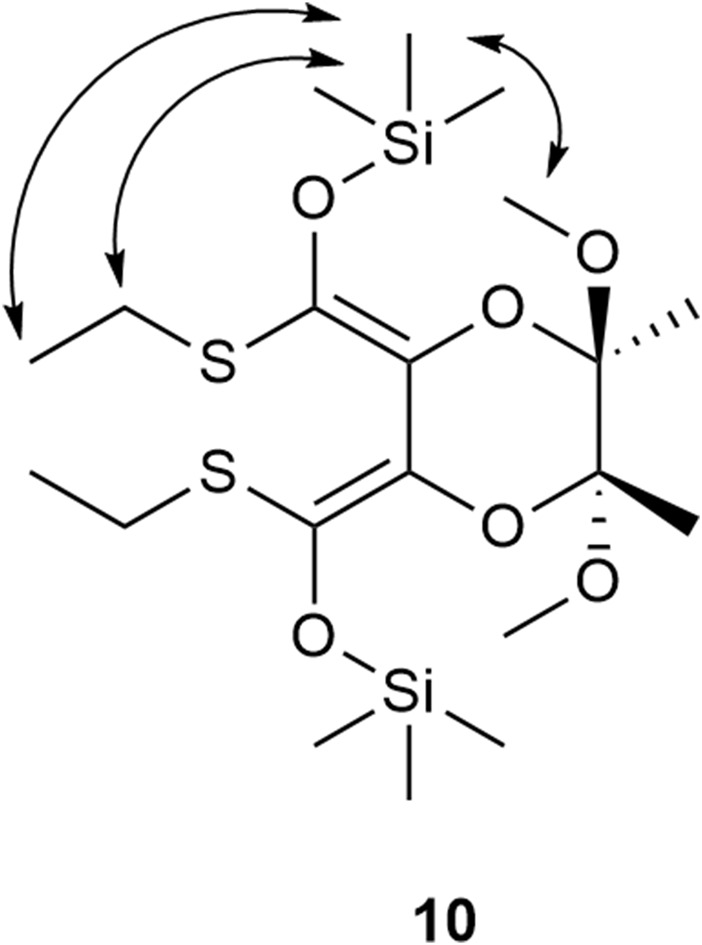
The structure of the silyl ether **10** and the interactions detected in the 2D NOE NMR spectrum.

To gain insight into the structure of the key intermediate, the dienolate generated from **2** was trapped with excess trimethylsilyl chloride (5 eq.TMSCl) to silyl enol ether derivatives (Figure 1A). The analysis of the crude reaction mixture by ^1^H NMR spectroscopy revealed the formation of a single predominant product (see Figure 1C). Subsequent 2D NMR experiments (NOESY, HSQC) facilitated the identification of a symmetrical bis(silyl-enol ether) **9** with a *Z*,*Z* configuration of double bonds (Figure 2).

**FIGURE 3:**
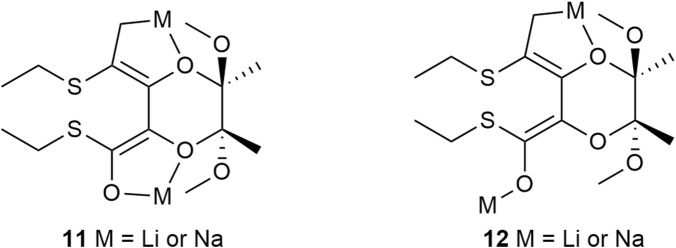
Proposed structures of dienolates **11** and **12**.

The results obtained with 1.1 and 2.2 eq. of LDA (Table 1, entry 1 and 2) demonstrate that the isomerization of (2*R*,3*R*,5*R*,6*R*)-2,3-butanediacetal protected dimethyl tartrate **2** to (2*R*,3*S*,5*R*,6*R*)-2,3-butanediacetal derivative **5** is more selective when both thioester groups are converted into enolate ions. In the derivative **5**, one thioester group is in the axial position and the second one is in the equatorial position. Consequently, this compound can only be obtained if the enolate ions in the dienolate intermediate differ; one hydrogen atom is attached axially and the other equatorially. This difference may be explained by different coordination of lithium/sodium ions - one may be coordinated by the oxygen atom of the 1,4-dioxane ring while the other remains uncoordinate and the orientation of the double bonds, which should not be coplanar. Because the enolate ion has the *Z*,*Z* geometry, it seems that lithium/sodium ions might be coordinated via the α-oxygen atoms of the BDA derivative **11** or **12** (Figure 3). This model of chelation was proposed for addition of zinc alkynylides to the equatorially located monoaldehyde BDA derivative (Shing et al., 2008). Whereas the β-chelation model can explain the stereoselectivity of addition of Grignard reagents to the axially positioned aldehyde of BDA (Leyva et al., 2008). When lithium/sodium ions from both enolate groups are coordinated by α-oxygen atoms the dienolate **11** has *C*_2_ symmetry (Figure 3). Protonation in this case may occur similarly at the axial positions of the carbon atoms C-2 and C-3 favoring the formation of the *trans*-product **2**. In contrast, if one oxygen atom from the 1,4-dioxane ring coordinates a metal ion **12** but the second lithium/sodium ion is not bound to the ring, the protons may be attached from the same side, leading to the formation of *cis*-isomer **5** (Figure 2).

**SCHEME 2:**
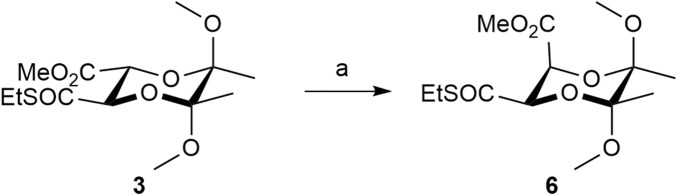
Isomerization of BDA derivatives **3**. Reagents: a) 1. 2.2 eq. of NaPTA, −78 °C, 2. MeOH.

Isomerization reactions using NaPTA as a base were also carried out for butanediacetal derivatives **1** and **3**. The isomerization reaction of (*2R*,*3R*,*5R*,*6R*)-5,6-dimethoxy-5,6-dimethyl-1,4-dioxane-2,3-dimethyl dicarboxylate **1** using NaPTA led to a mixture of compounds: **1**, **4**, **7**, and **8** in the ratio: 1:0.9:2.4:1.2. The ratio of the products obtained is similar to the reaction using LDA described in the literature (Barros et al., 1999). However, for the mixed ester-thioester **3**, the reaction with NaPTA proceeded with excellent selectivity, yielding the *cis*-product **6** in over 90% yield (Scheme 2). This matches the performance of LDA and demonstrates that NaPTA is a powerful and practical alternative for isomerization of thioester-containing BDAs.

## 4 Conclusion

We developed efficient stereoselective isomerization protocols for 2,3-disubstituted butanediacetal derivatives. Using LDA, stereoselectivity increased dramatically upon addition of 6 equiv LiCl, yielding a *cis*:*trans* ratio of 5.8:1. More importantly, NaPTA enabled comparable selectivity (6:1) without additives and without formation of β-elimination product. This development represents a practical alternative to LDA. Mechanistic considerations, including the trapping of the reaction intermediate as a *Z*,*Z*-bis(silyl enol ether), indicated that high stereoselectivity is controlled by the formation of an asymmetric dienolate structure. NaPTA also showed excellent selectivity for mixed ester–thioester BDAs. These results establish NaPTA as a powerful tool for stereocontrolled isomerization of BDA derivatives and expand the available methodology for asymmetric enolate chemistry.

## Data Availability

The original contributions presented in the study are included in the article/Supplementary Material, further inquiries can be directed to the corresponding author.
