# Supplementary material for: Selective isomerization of 2,3-disubstituted butanediacetal esters and thioesters using lithium and sodium enolates: effects of bases and additives on stereoselectivity
Source: Front Chem. 2026 Feb 4;14:1750600. doi: 10.3389/fchem.2026.1750600 (PMC12913133; doi:10.3389/fchem.2026.1750600)
Supplement: Supplementary file 1 [file DataSheet1.pdf]

Supplementary Material

Selective Isomerization of 2,3-Disubstituted Butanediacetal Esters and Thioesters Using Lithium and Sodium Enolates: Effects of Bases and Additives on Stereoselectivity.

Adam Drop, Magdalena Grzegolec and Bożena Frąckowiak-Wojtasek*

Faculty of Chemistry
Opole University
ul. Oleska 48
45-052 Opole
Poland

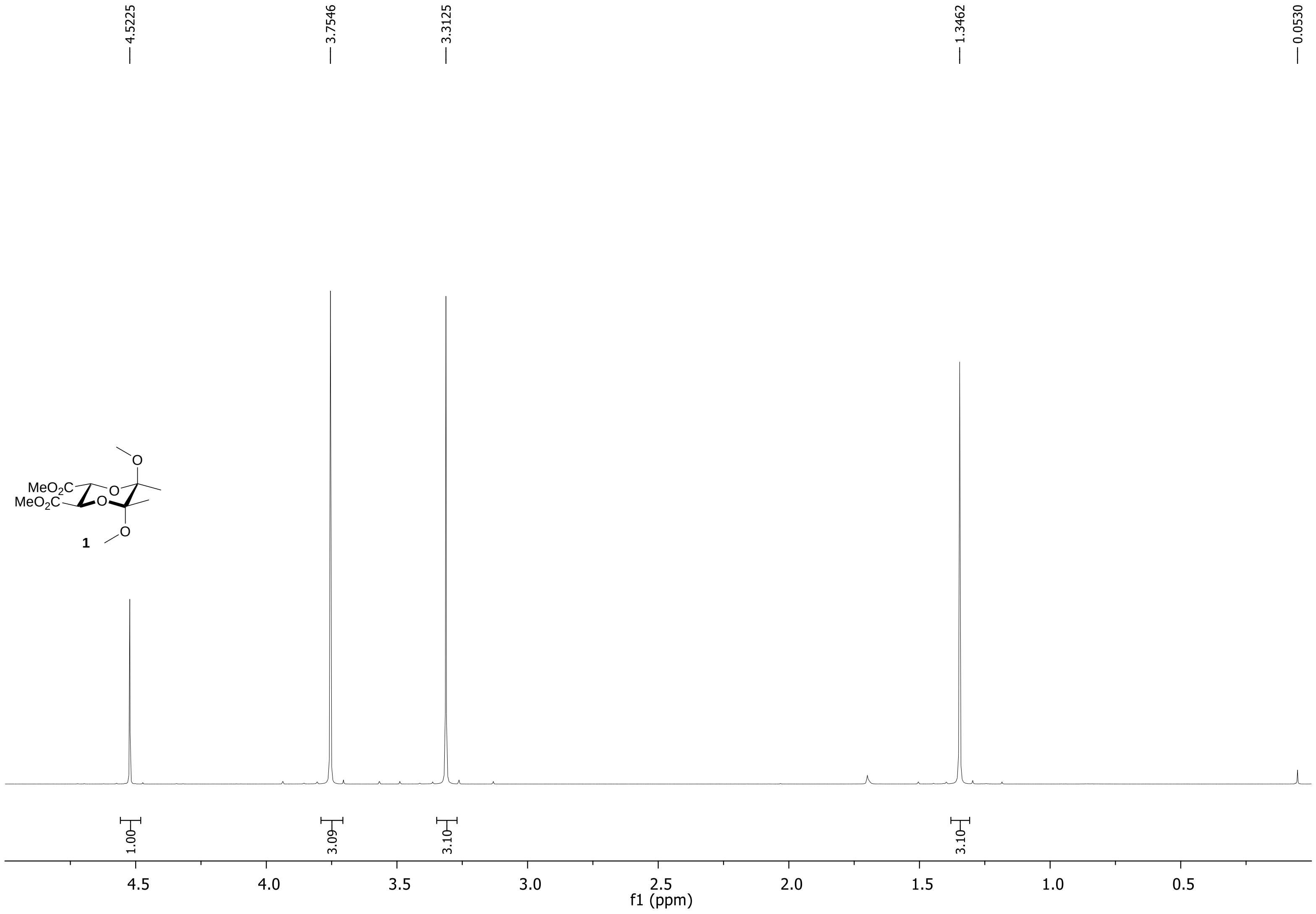

Figure S1 ^{1}H NMR spectrum of (2*R*,3*R*,5*R*,6*R*)-5,6-dimethoxy-5,6-dimethyl-1,4-dioxane-2,3-dimethyl dicarboxylate **1**.

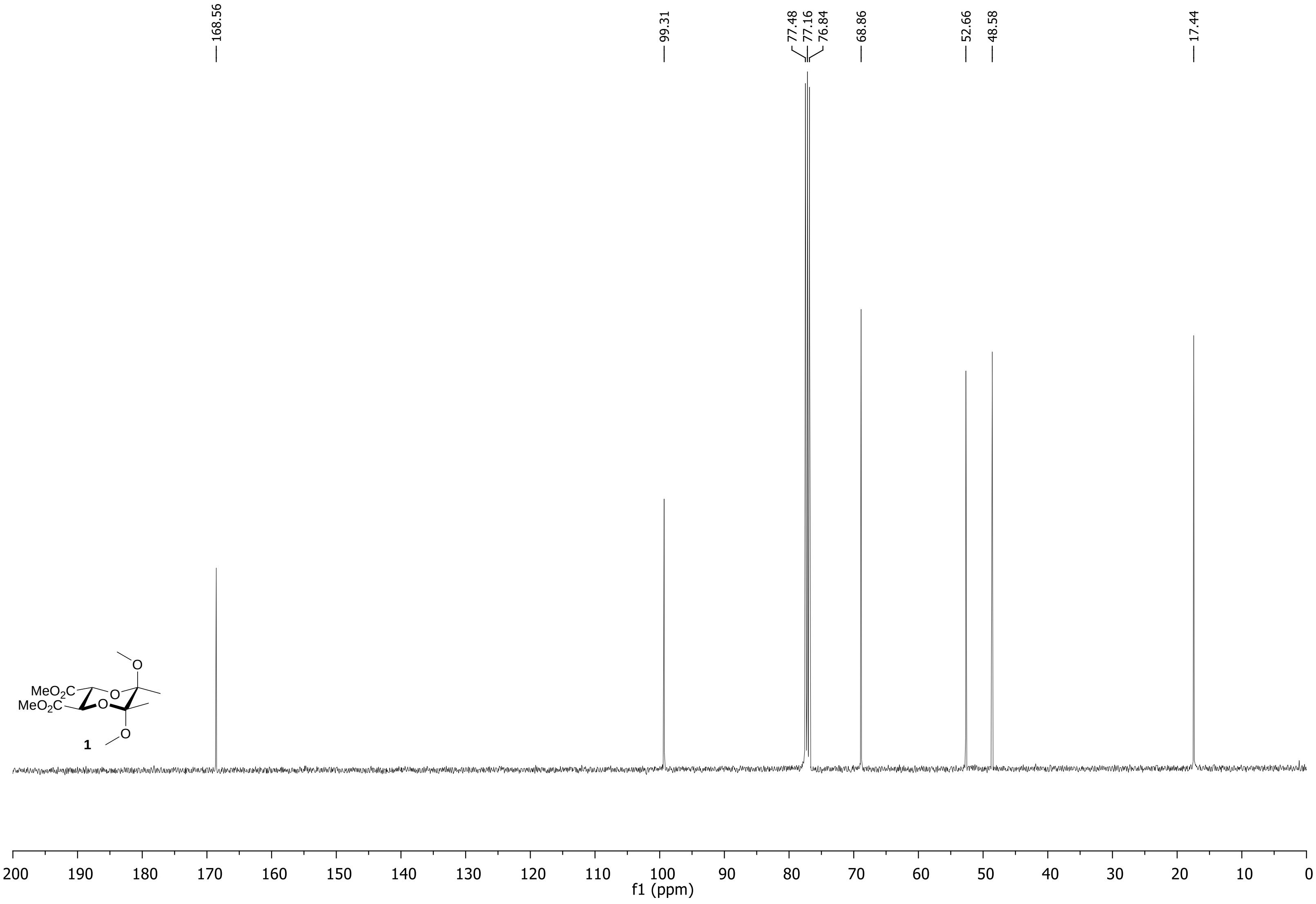

Figure S2 ^{13}C NMR spectrum of (2*R*,3*R*,5*R*,6*R*)-5,6-dimethoxy-5,6-dimethyl-1,4-dioxane-2,3-dimethyl dicarboxylate **1**.

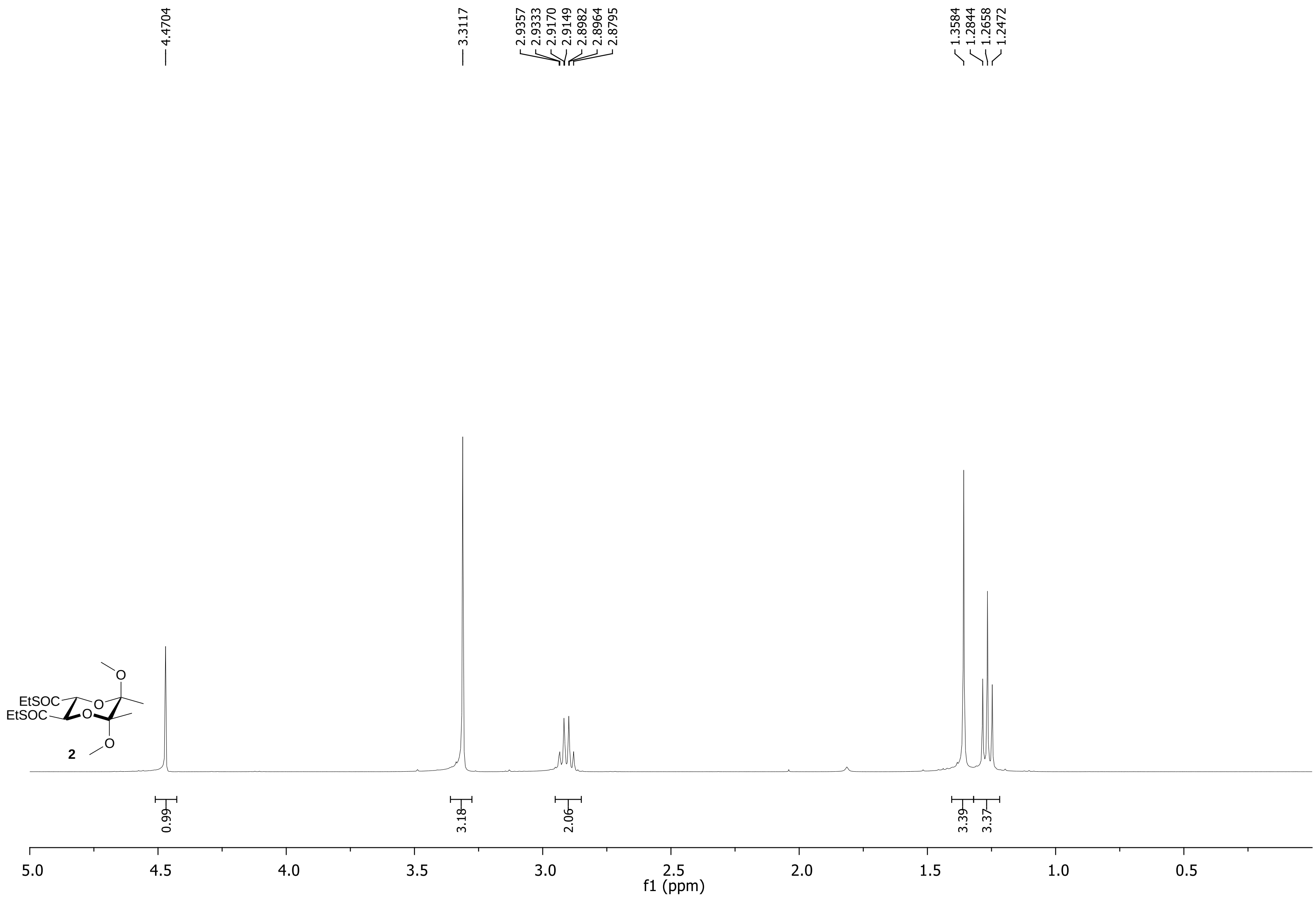

Figure S3 ^{1}H NMR spectrum of S2,S3-diethyl (2*R*,3*R*,5*R*,6*R*)-5,6-dimethoxy-5,6-dimethyl-1,4-dioxane-2,3-dicarbothioate **2**.

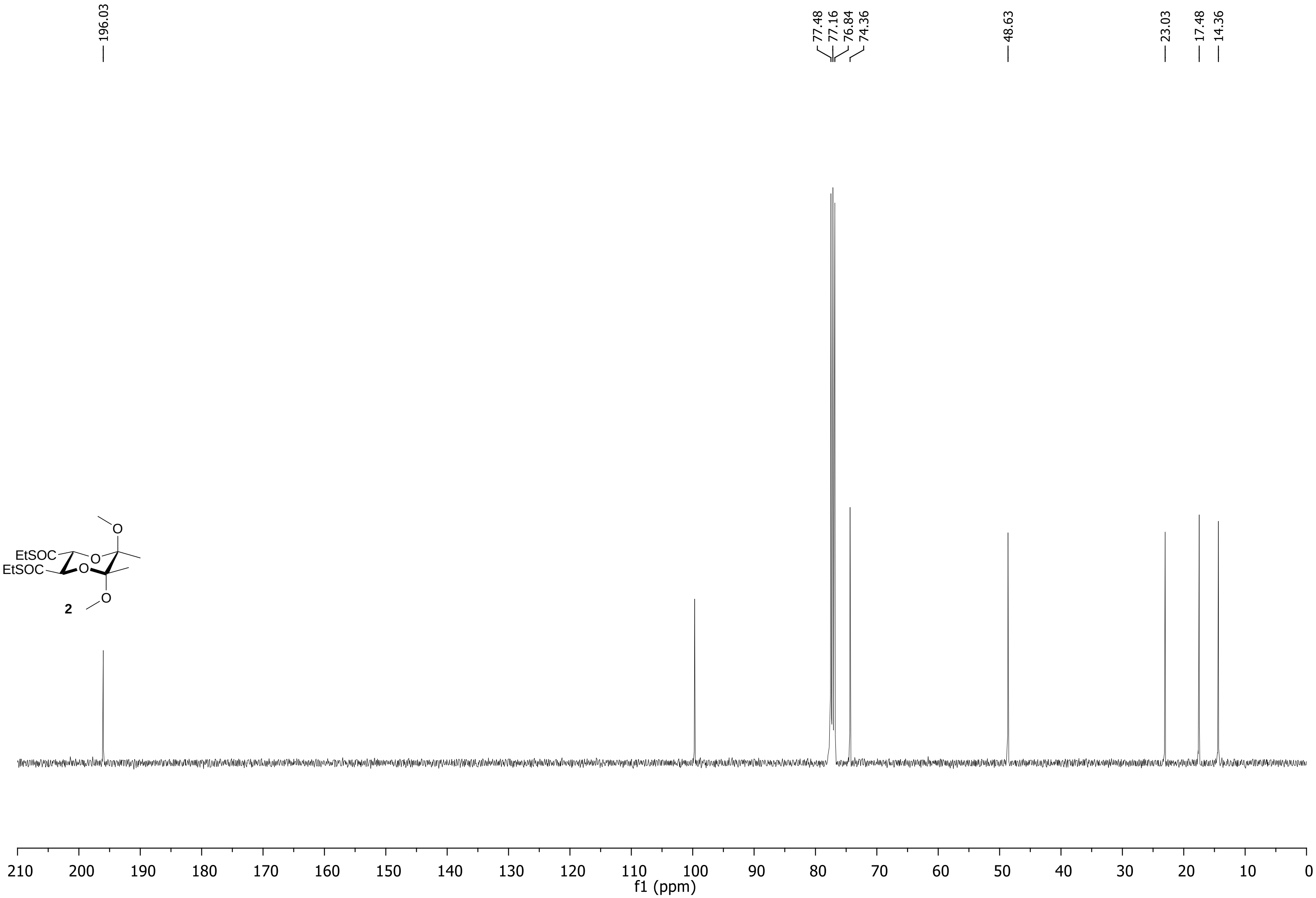

Figure S4 ^{13}C NMR spectrum of S2,S3-diethyl (2*R*,3*R*,5*R*,6*R*)-5,6-dimethoxy-5,6-dimethyl-1,4-dioxane-2,3-dicarbothioate **2**.

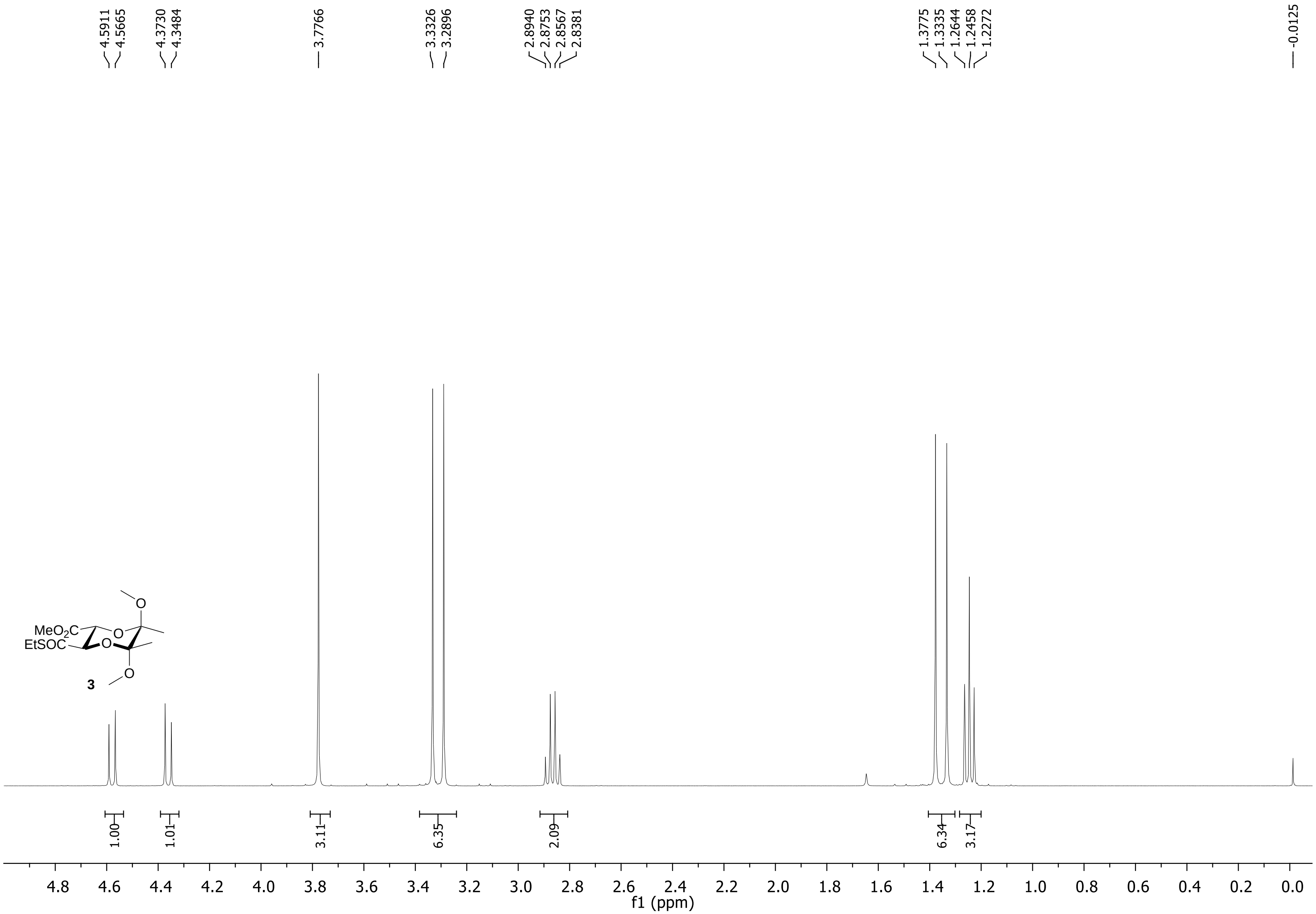

Figure S5 ^{1}H NMR spectrum of methyl (2*R*,3*R*,5*R*,6*R*)-3-ethylsulfanylcarbonyl-5,6-dimethoxy-5,6-dimethyl-1,4-dioxane-2-carboxylate **3**.

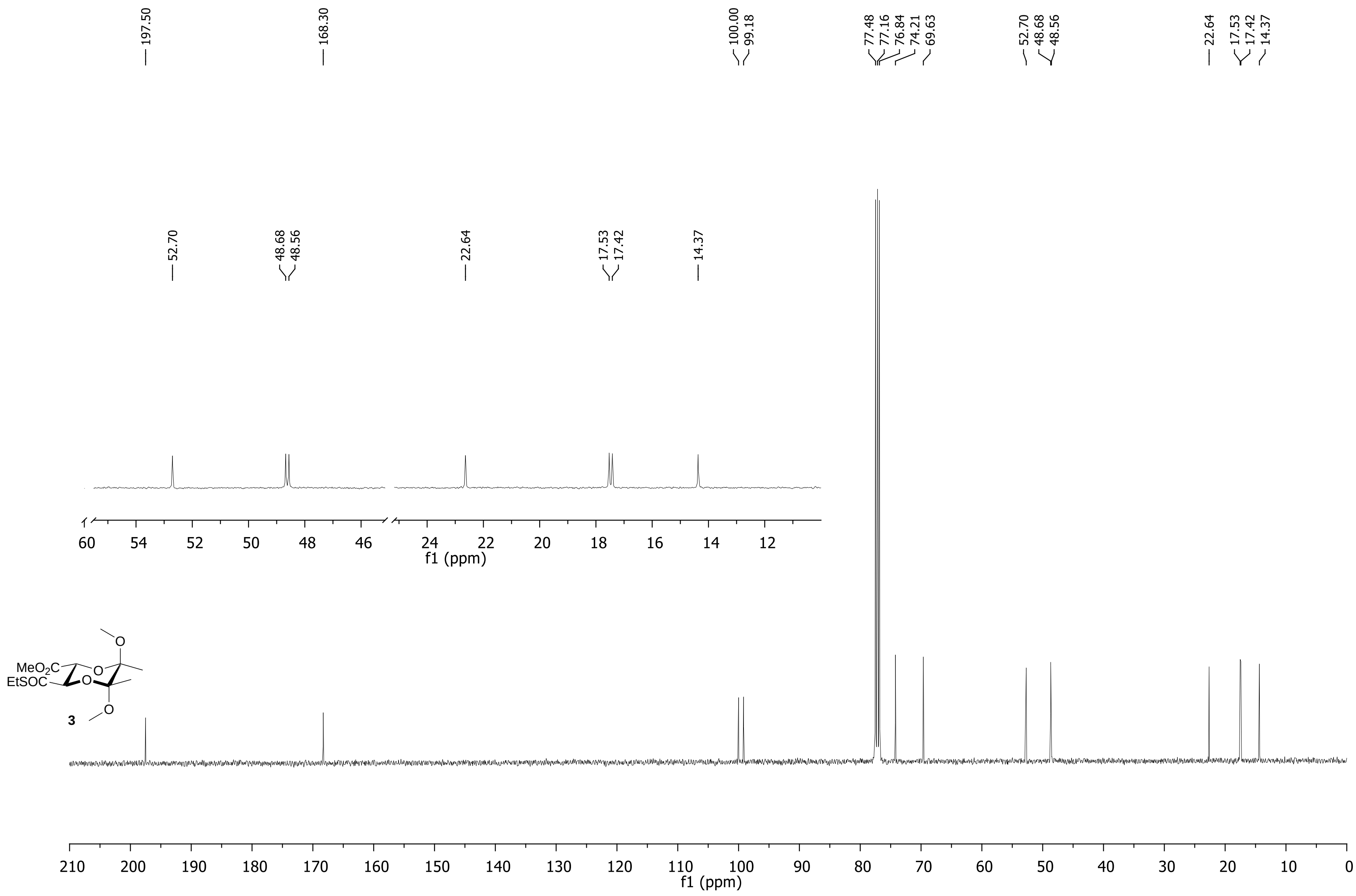

Figure S6 ^{13}C NMR spectrum of methyl (2*R*,3*R*,5*R*,6*R*)-3-ethylsulfanylcarbonyl-5,6-dimethoxy-5,6-dimethyl-1,4-dioxane-2-carboxylate **3**.

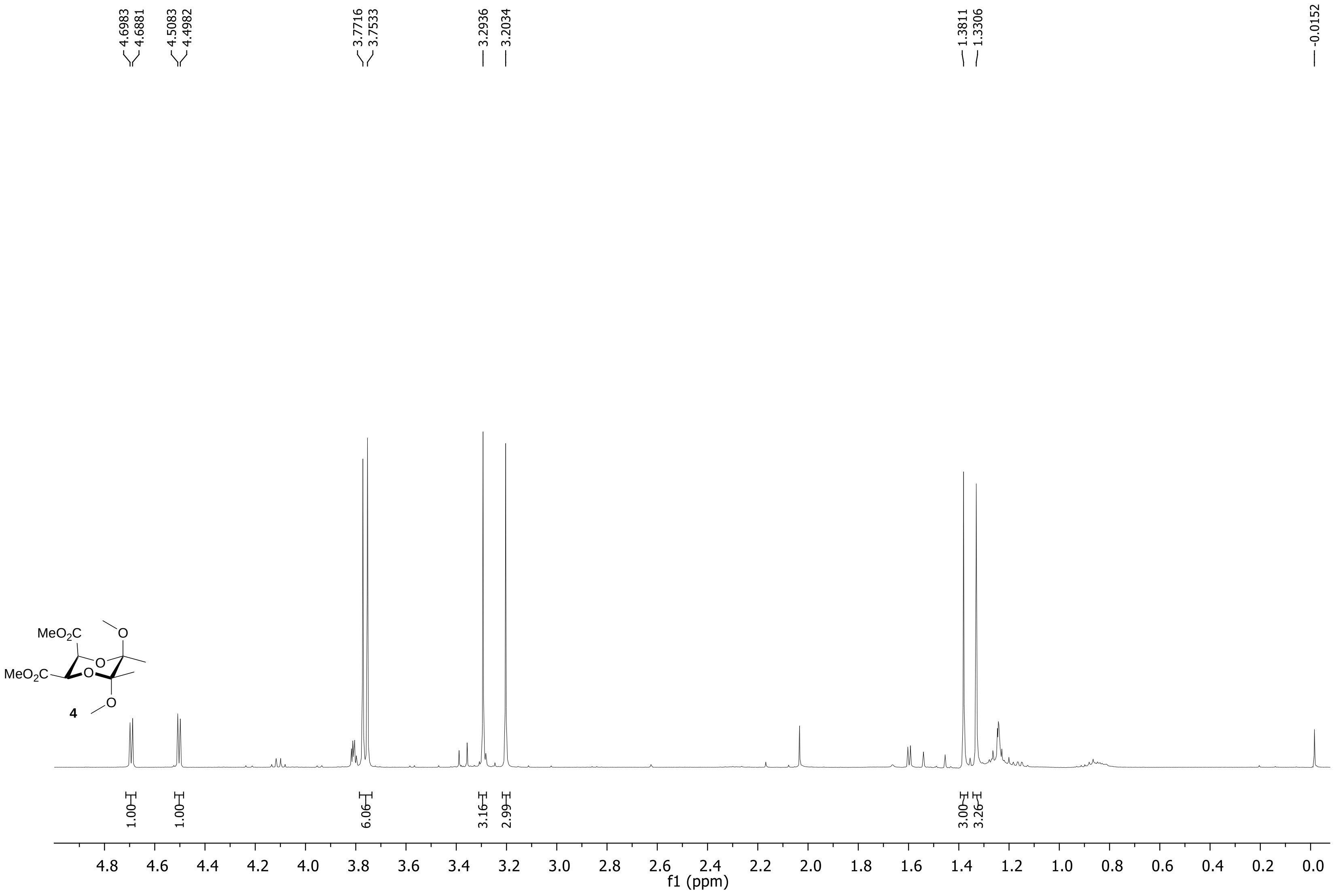

Figure S7 ^{1}H NMR spectrum of (2*R*,3*S*,5*R*,6*R*)-5,6-dimethoxy-5,6-dimethyl-1,4-dioxane-2,3-dimethyl dicarboxylate **4**.

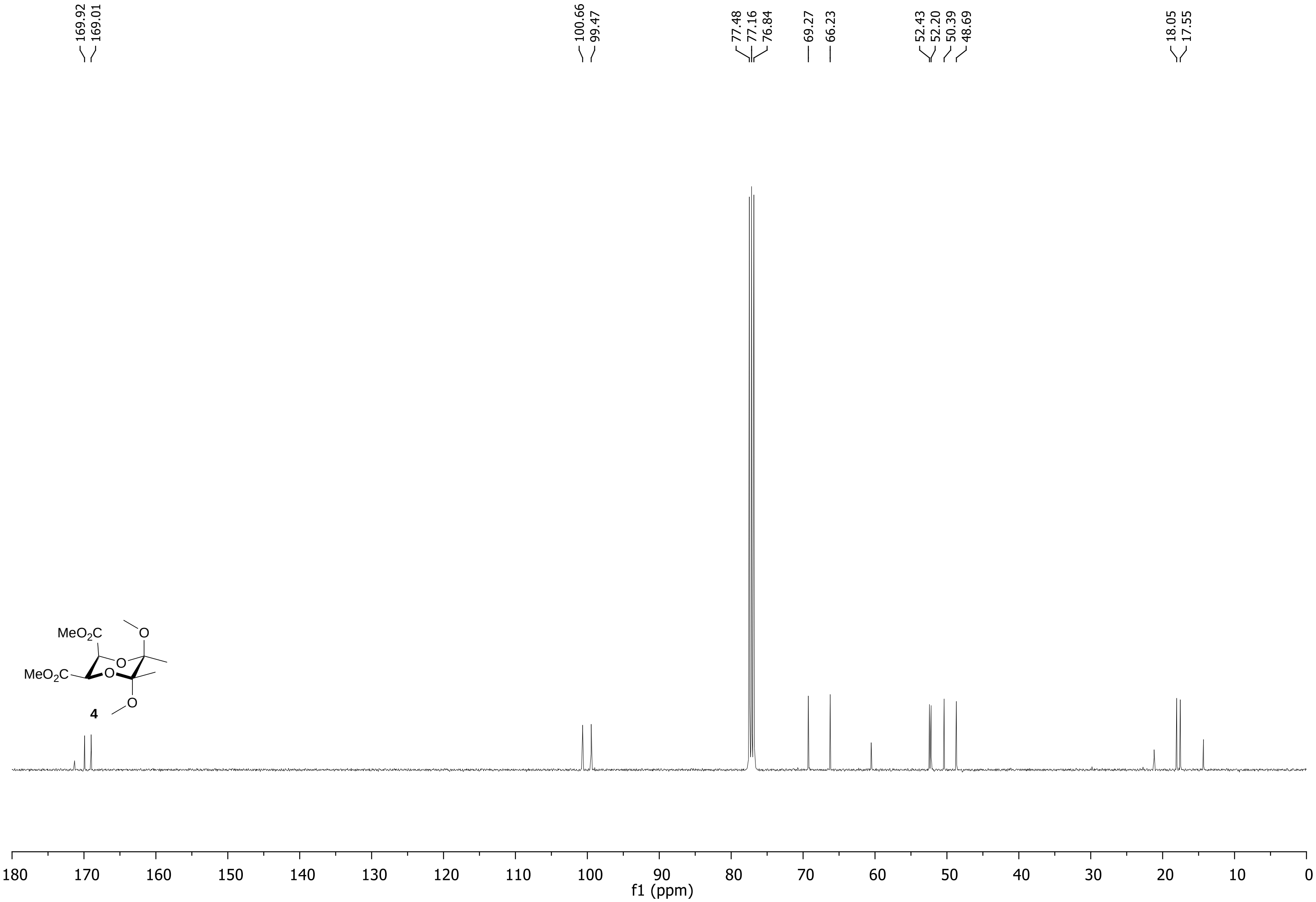

Figure S8 ^{13}C NMR spectrum of (2*R*,3*S*,5*R*,6*R*)-5,6-dimethoxy-5,6-dimethyl-1,4-dioxane-2,3-dimethyl dicarboxylate **4**.

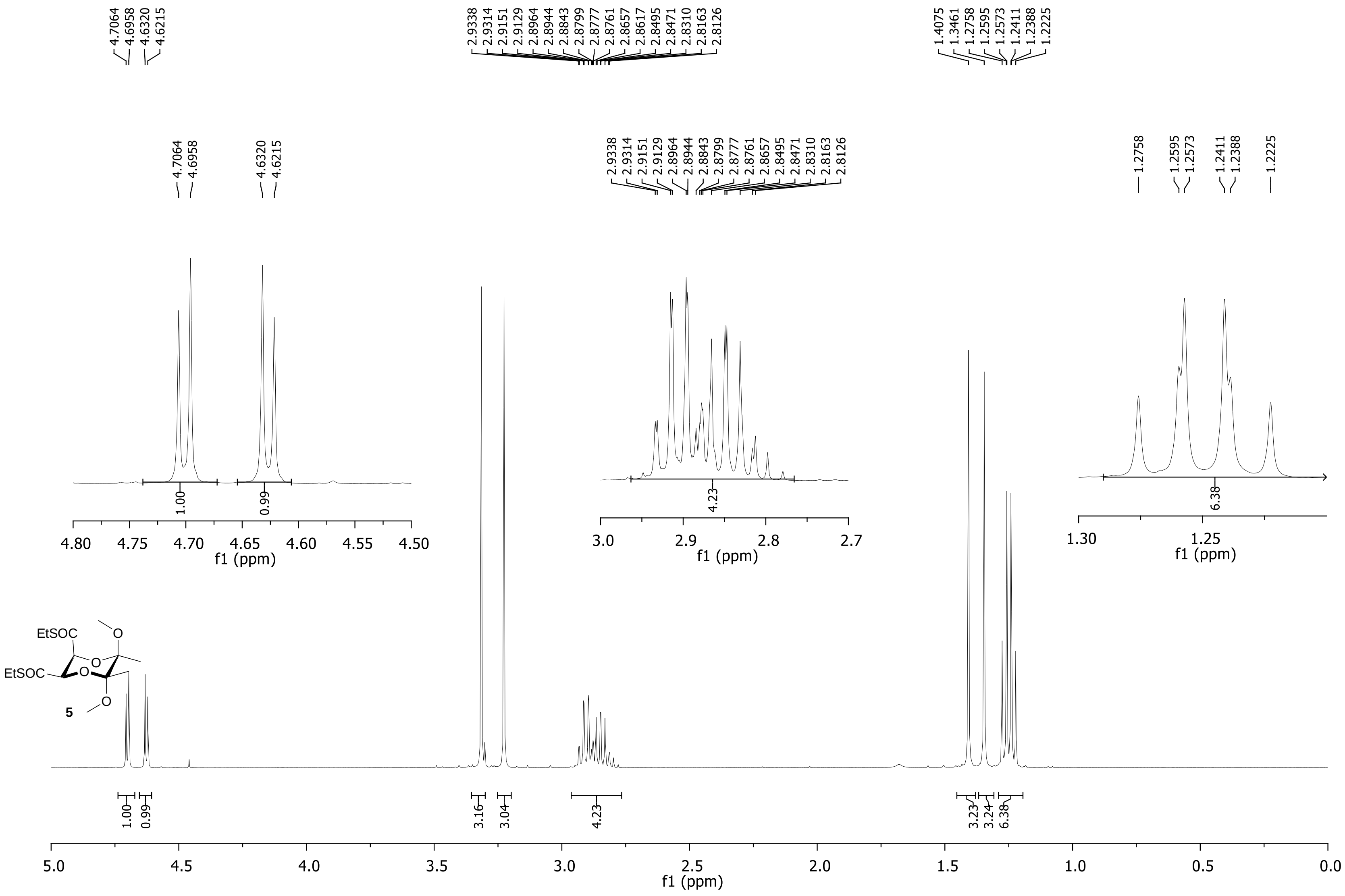

Figure S9 ^{1}H NMR spectrum of S2,S3-diethyl (2*R*,3*S*,5*R*,6*R*)-5,6-dimethoxy-5,6-dimethyl-1,4-dioxane-2,3-dica*R*bothioate **5**.

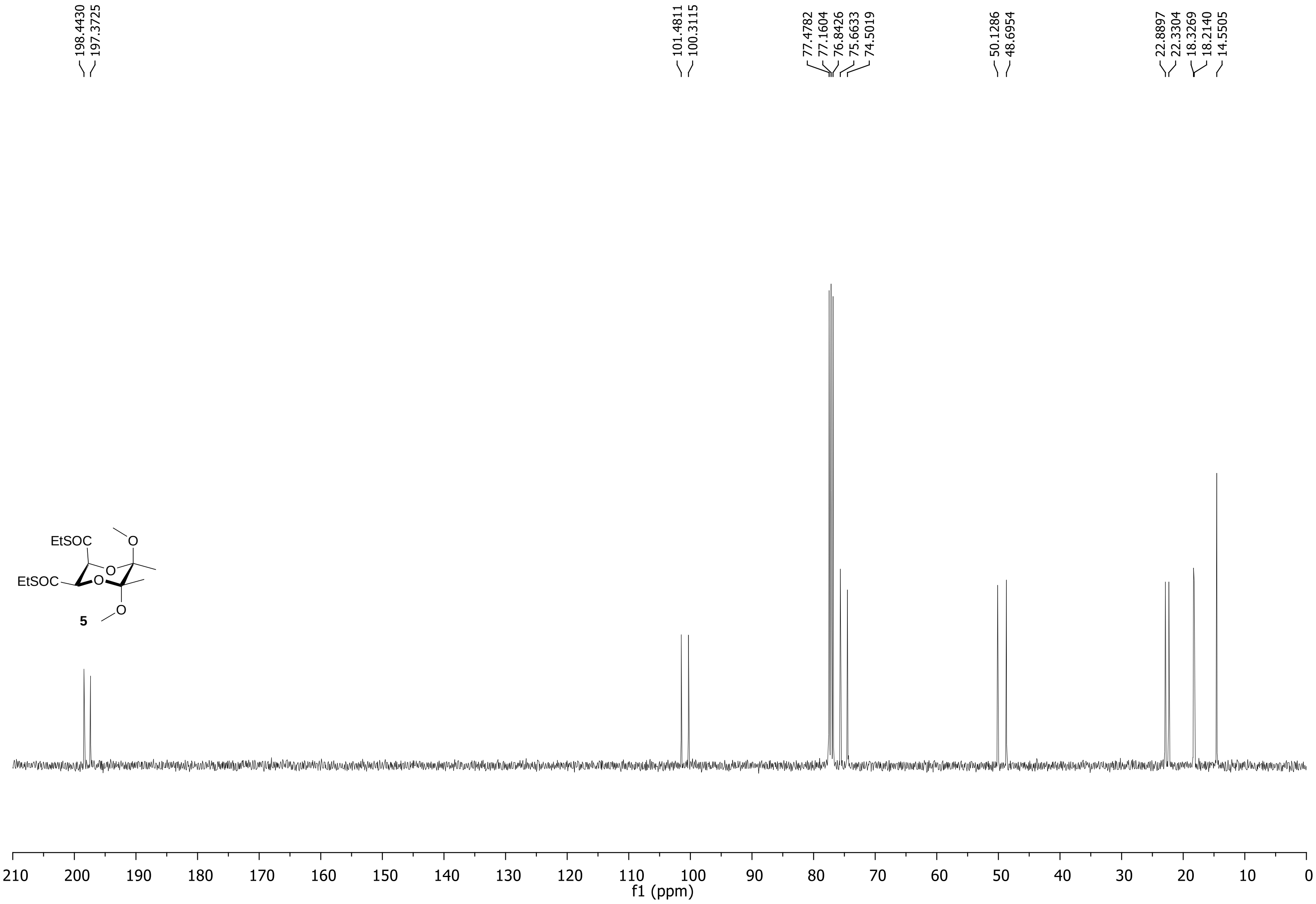

Figure S10 ^{13}C NMR spectrum of S2,S3-diethyl (2*R*,3*S*,5*R*,6*R*)-5,6-dimethoxy-5,6-dimethyl-1,4-dioxane-2,3-dicarbothioate **5**.

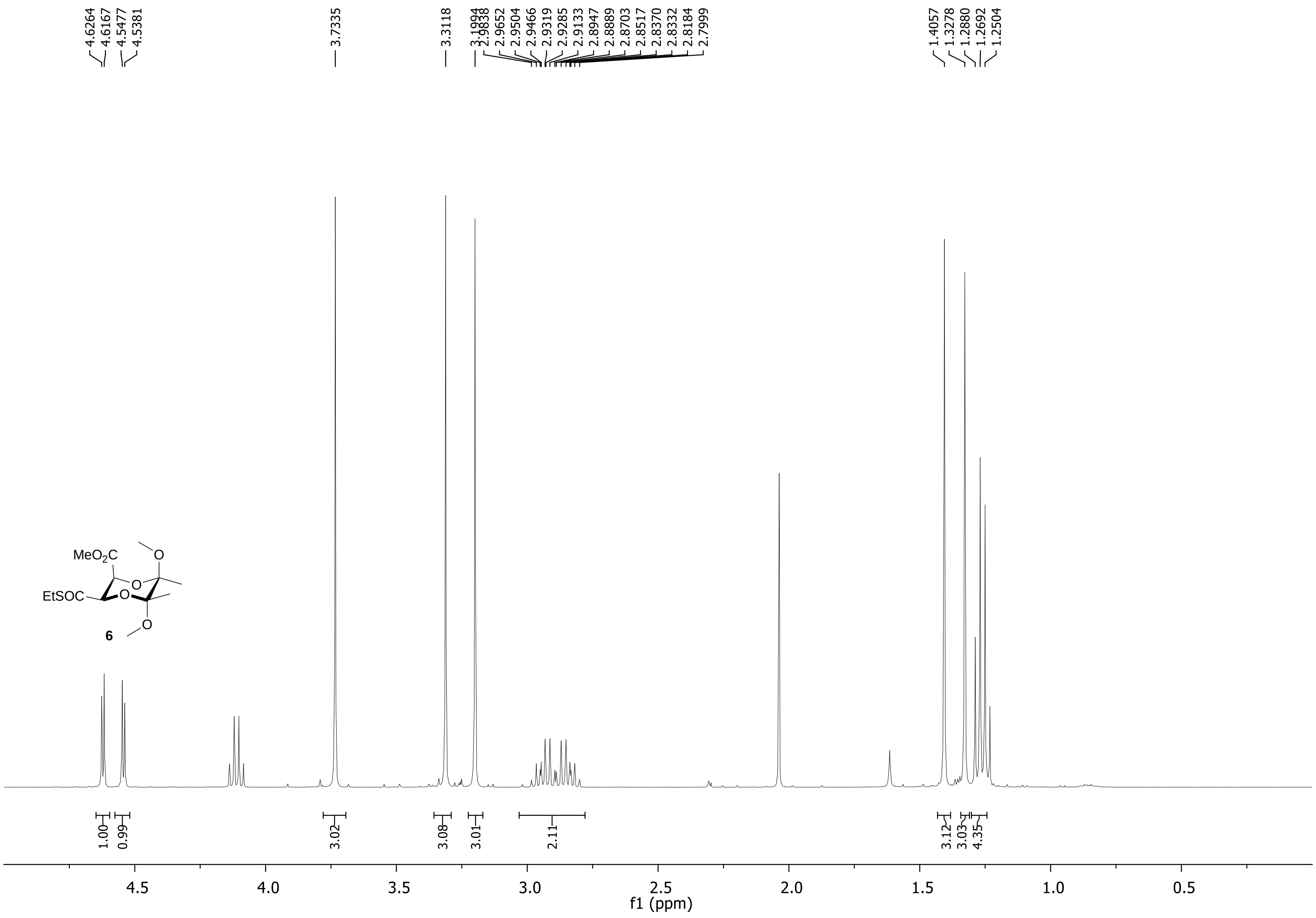

Figure S11 ^{1}H NMR spectrum of methyl (2*S*,3*R*,5*R*,6*R*)-3-ethylsulfanylcarbonyl-5,6-dimethoxy-5,6-dimethyl-1,4-dioxane-2-carboxylate **6** in the addition of ethyl acetate.

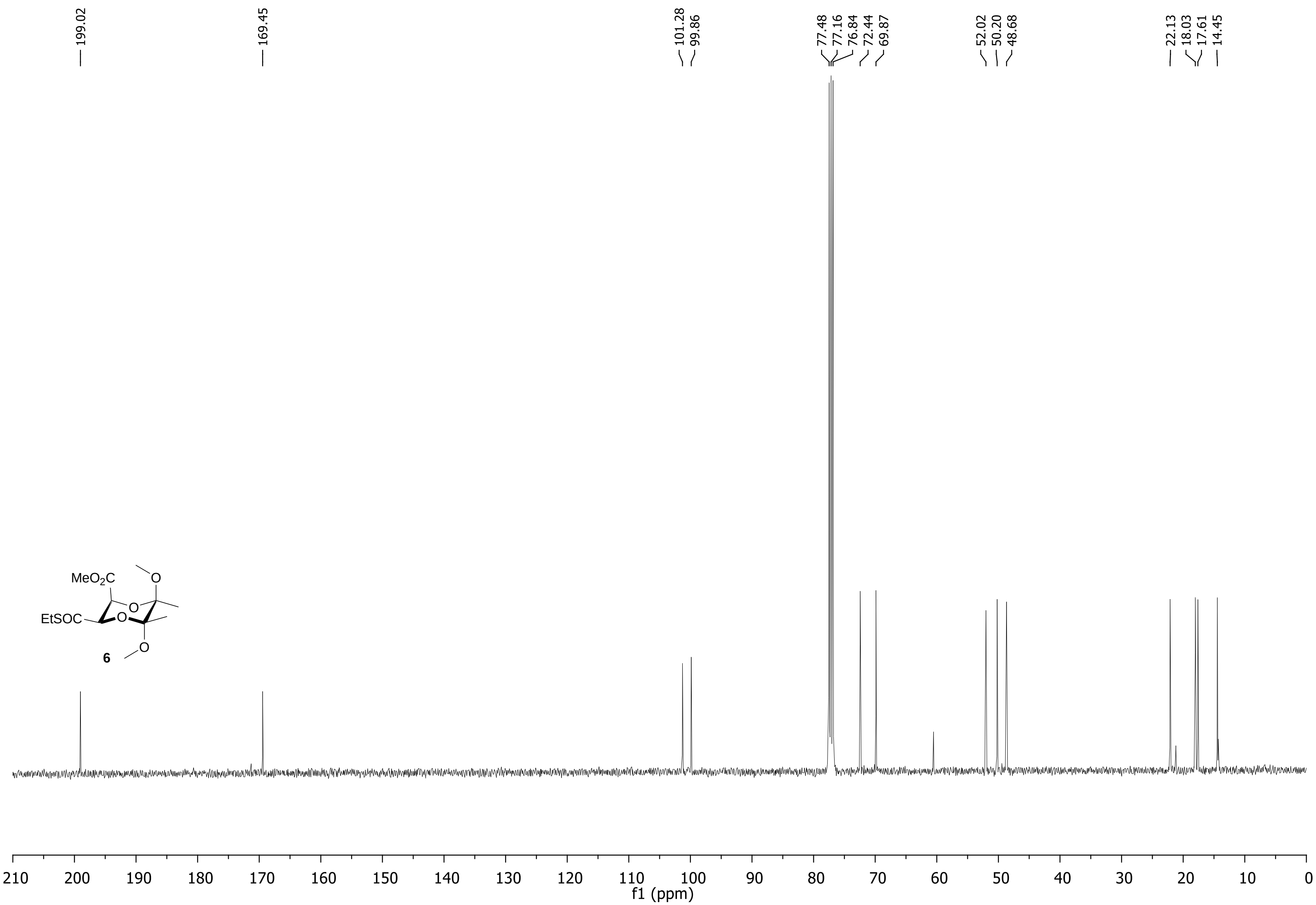

Figure S12 ^13^C NMR spectrum of methyl (2*S*,3*R*,5*R*,6*R*)-3-ethylsulfanylcarbonyl-5,6-dimethoxy-5,6-dimethyl-1,4-dioxane-2-carboxylate **6**.

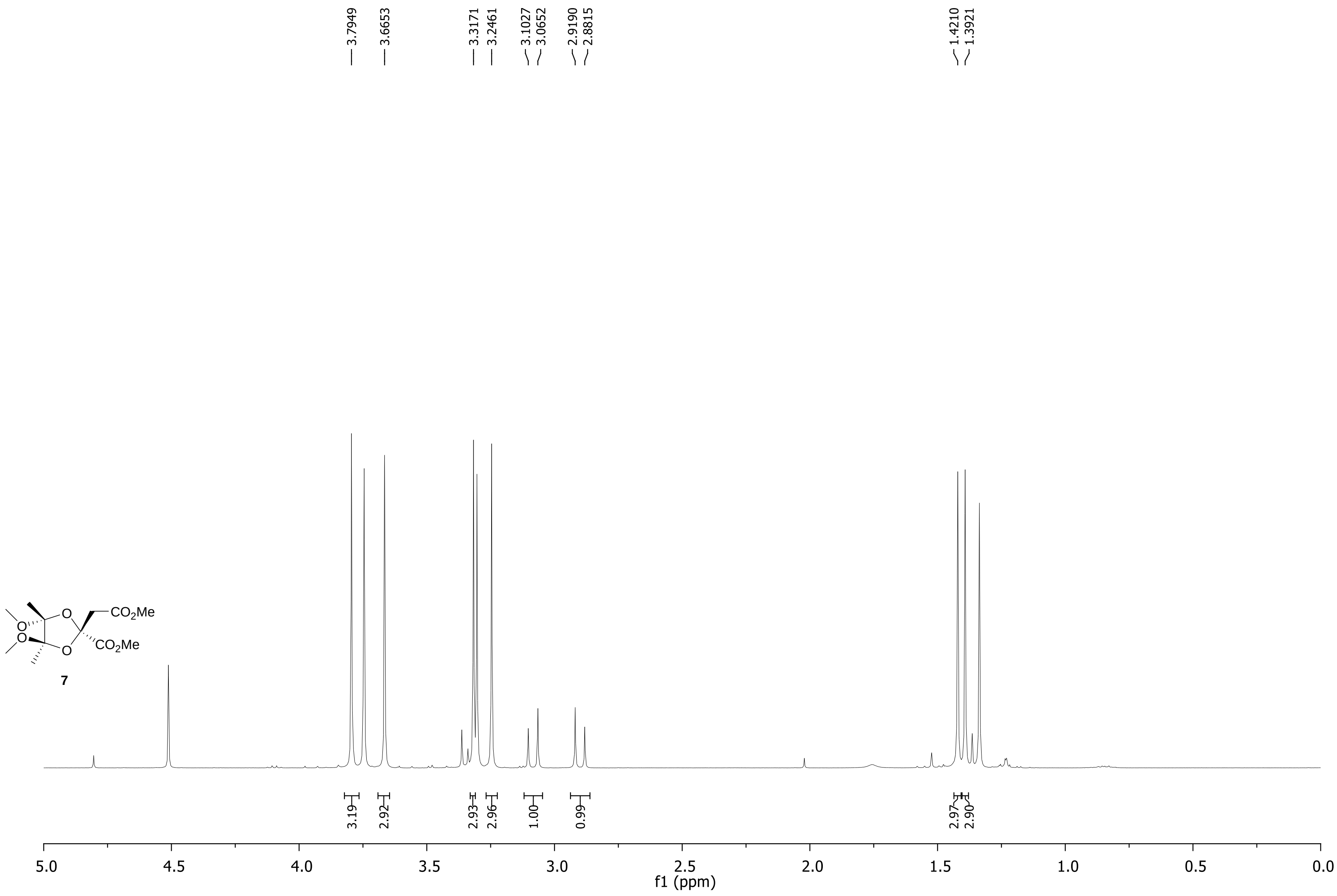

Figure S13 ^{1}H NMR spectrum of methyl (4*R*,5*R*)-4,5-dimethoxy-4,5-dimethyl -2-(2-methoxy-2-oxoethyl)-1,3-dioxolane-2-carboxylate **7** in the mixture with **1**.

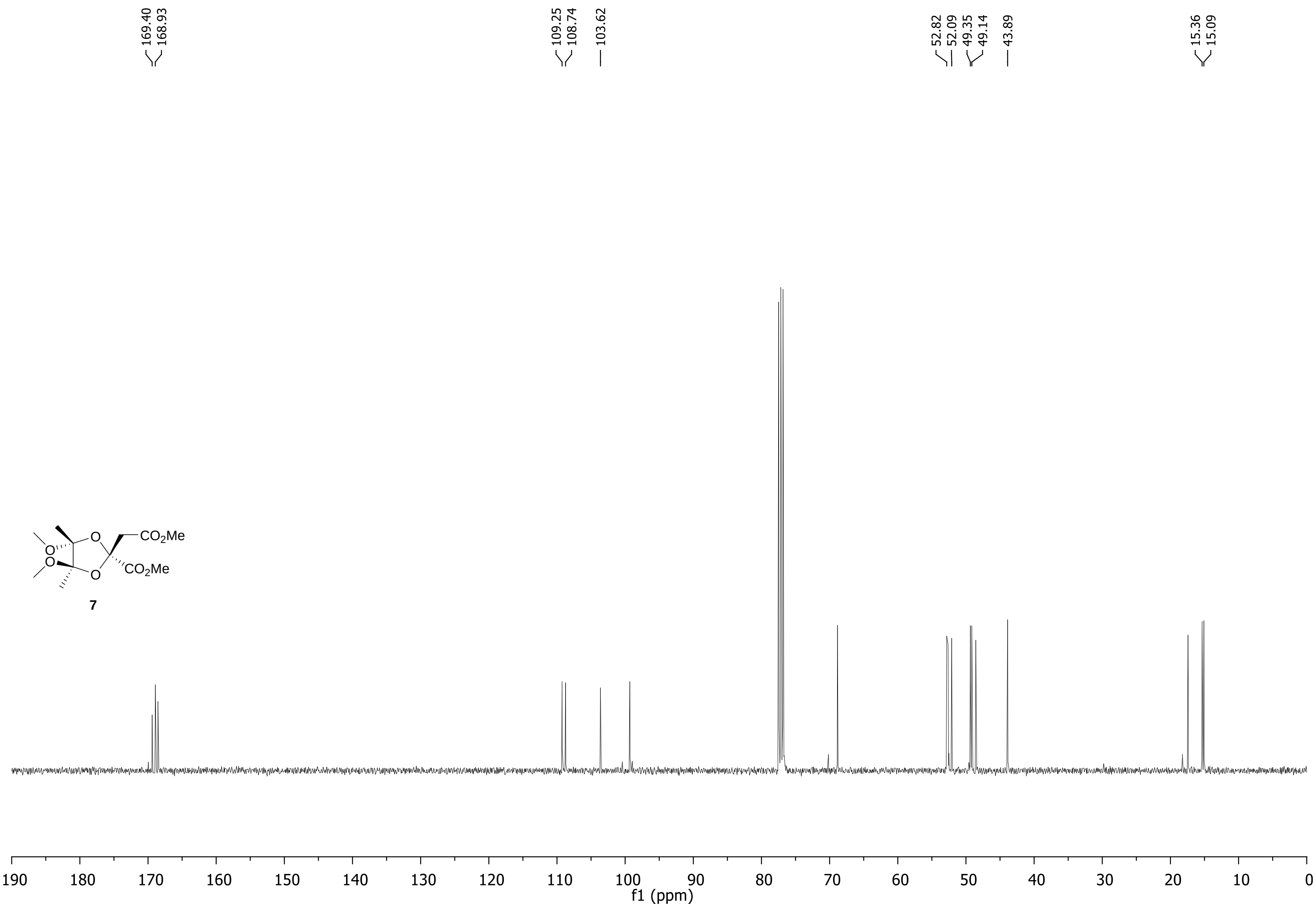

Figure S14 ^{13}C NMR spectrum of methyl (4*R*,5*R*)-4,5-dimethoxy-4,5-dimethyl -2-(2-methoxy-2-oxoethyl)-1,3-dioxolane-2-carboxylate **7** in the mixture with **1**.

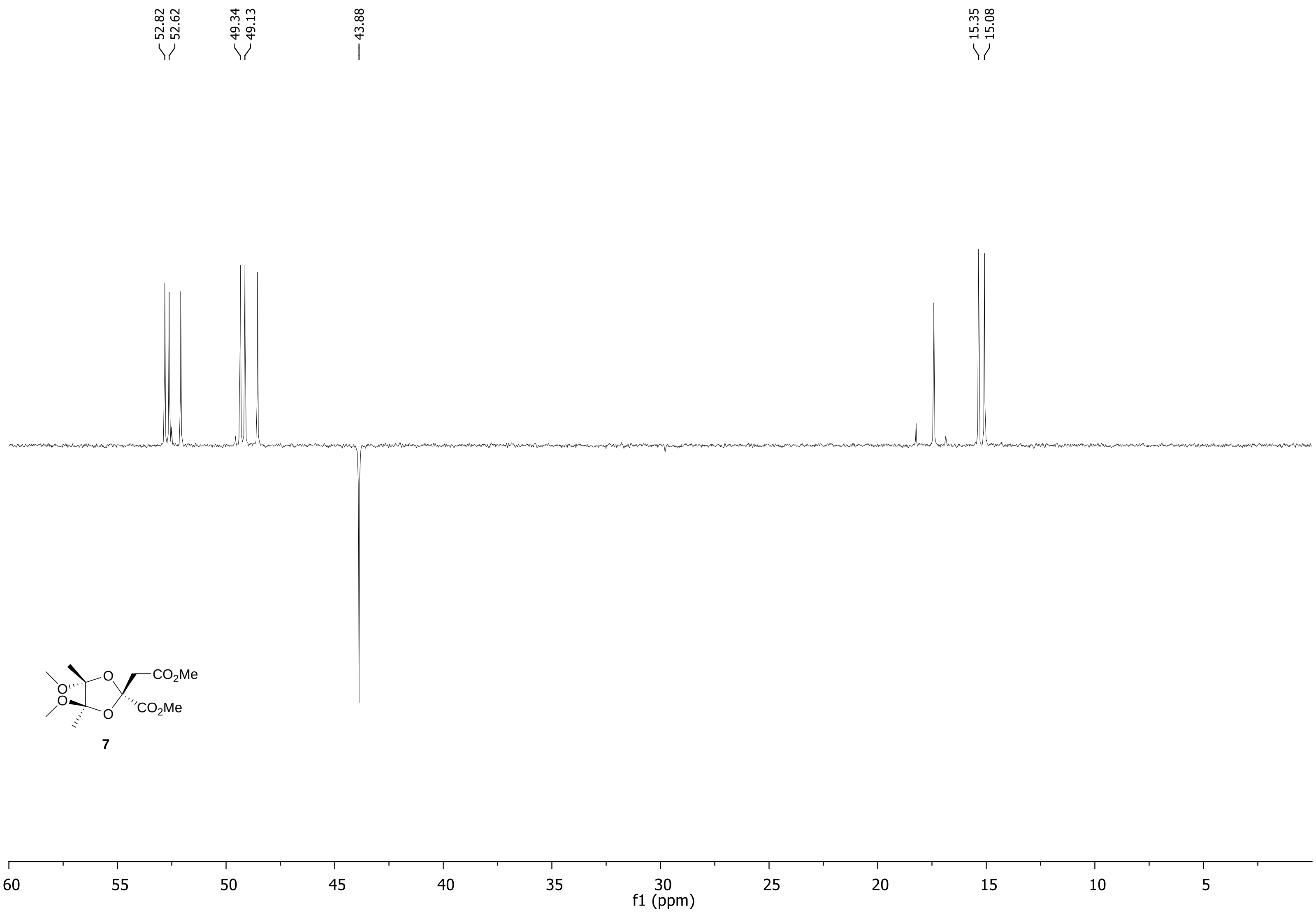

Figure S15 dept135 ^{13}C NMR spectrum of methyl (4*R*,5*R*)-4,5-dimethoxy-4,5-dimethyl -2-(2-methoxy-2-oxoethyl)-1,3-dioxolane-2-carboxylate **7** in the mixture with **1**.

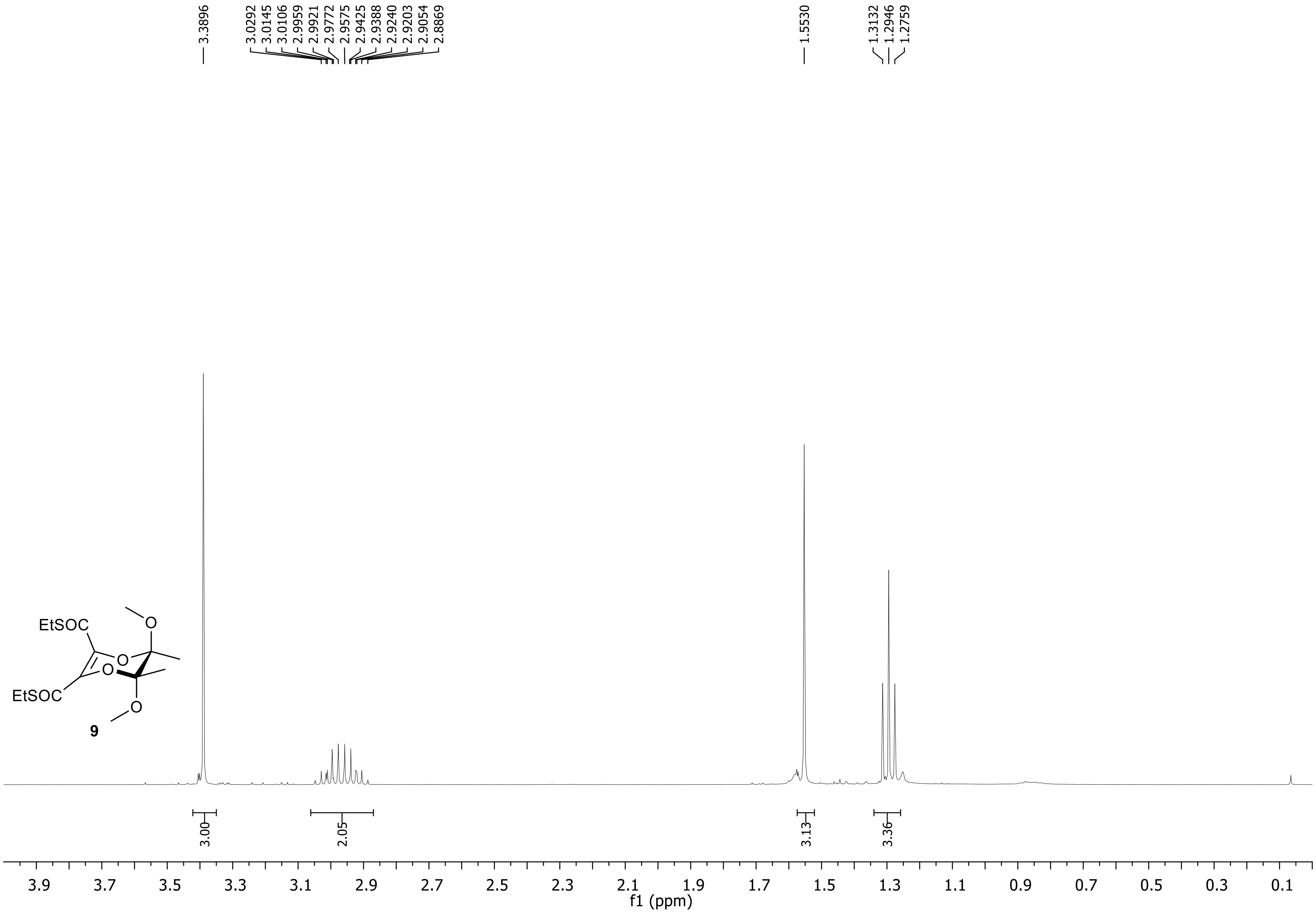

Figure S15 ^{1}H NMR spectrum of **9**.

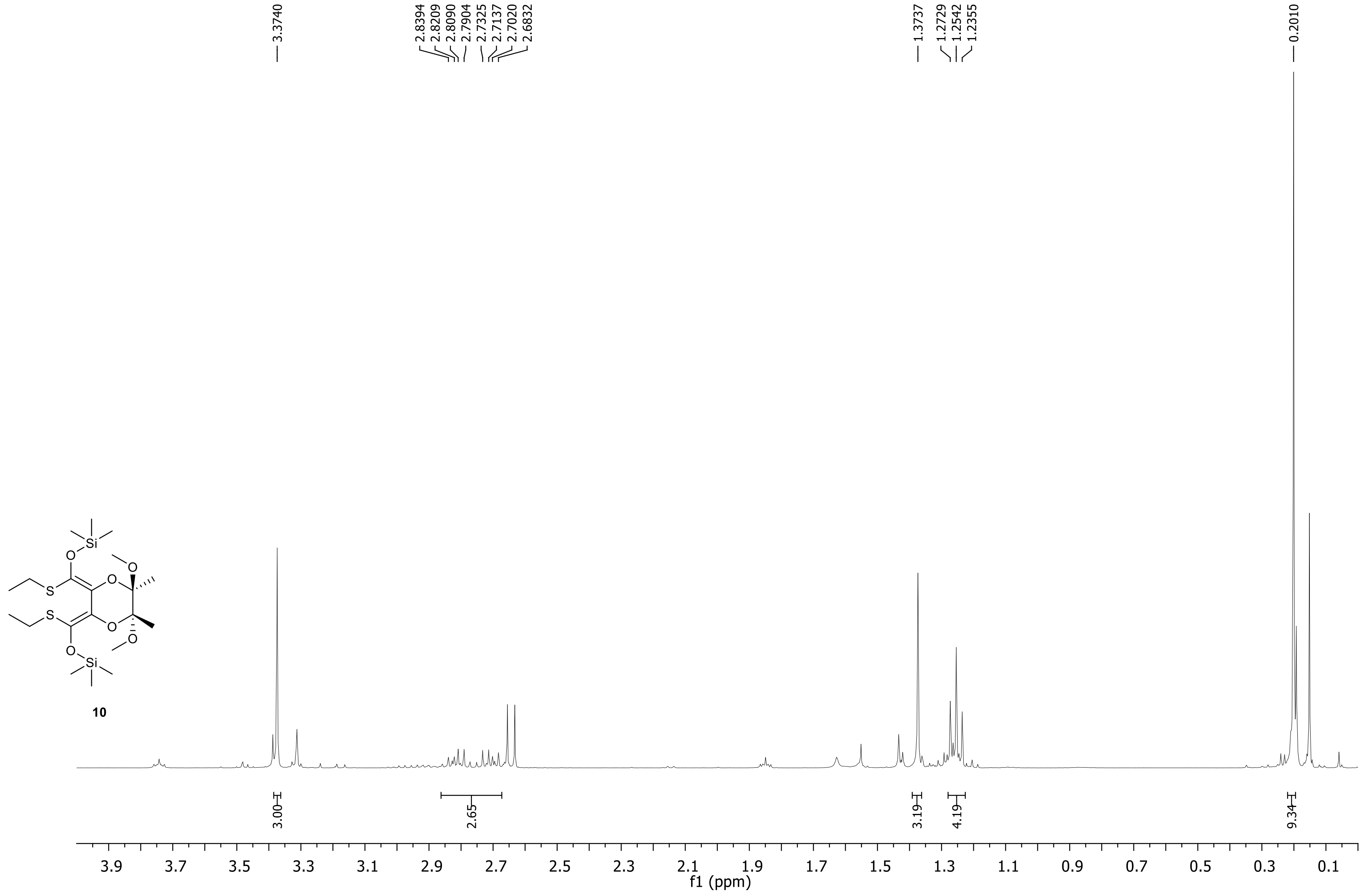

Figure S16 ^{1}H NMR spectrum of **10**.

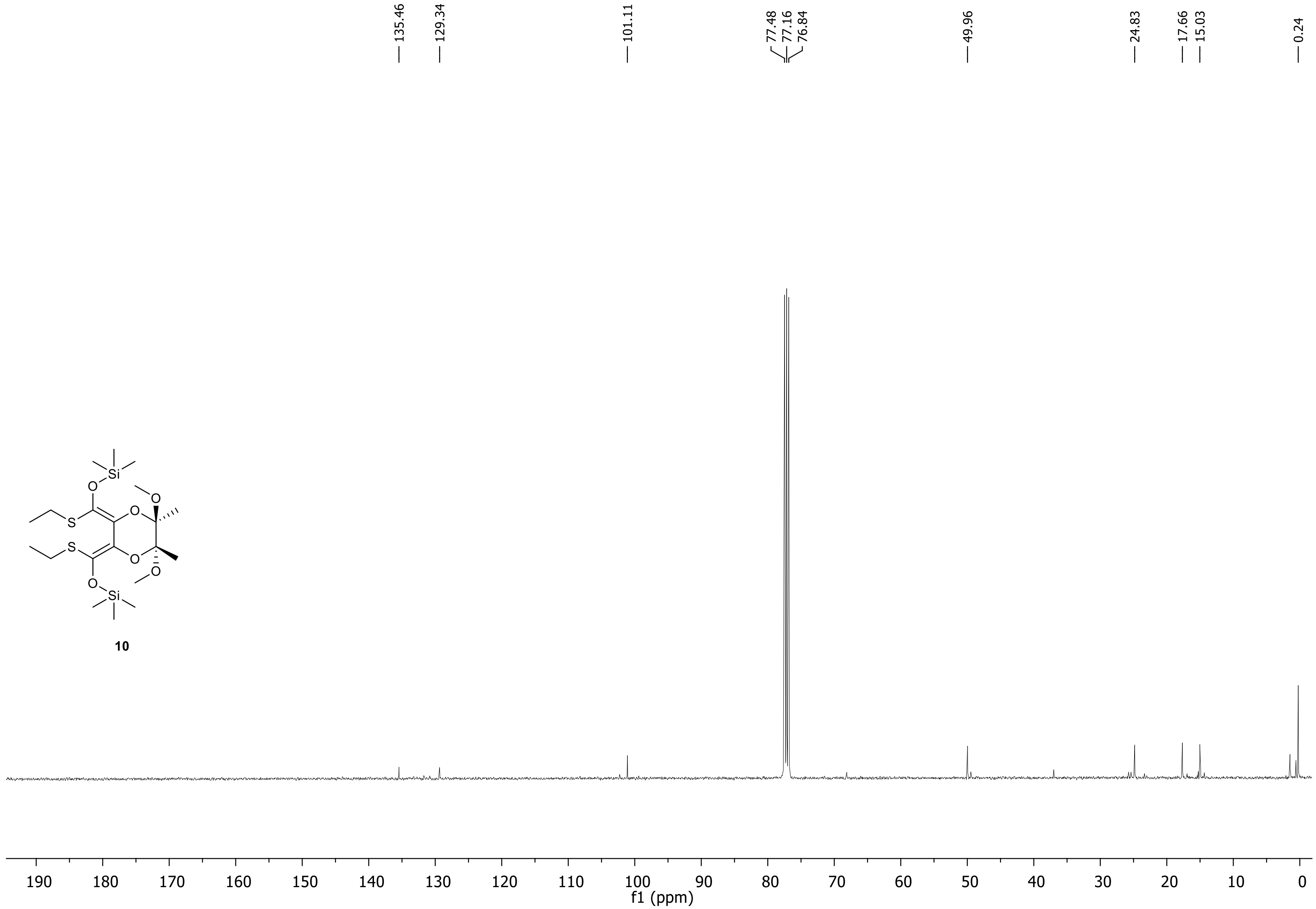

Figure S17 ^{13}C NMR spectrum of **10**.

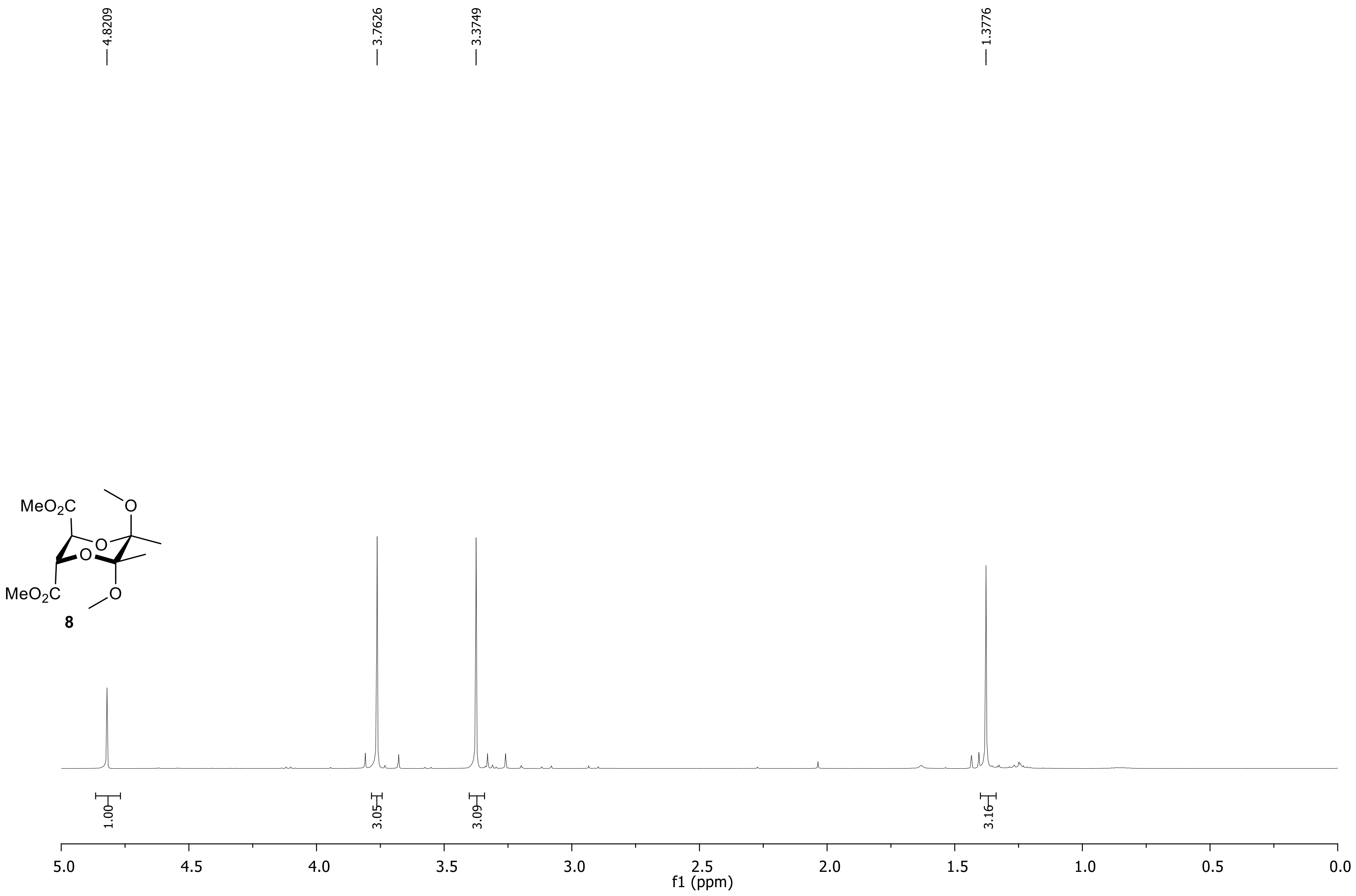

Figure S18 ^{1}H NMR spectrum of (2*S*,3*S*,5*R*,6*R*)-5,6-dimethoxy-5,6-dimethyl-1,4-dioxane-2,3-dimethyl dicarboxylate **8**.

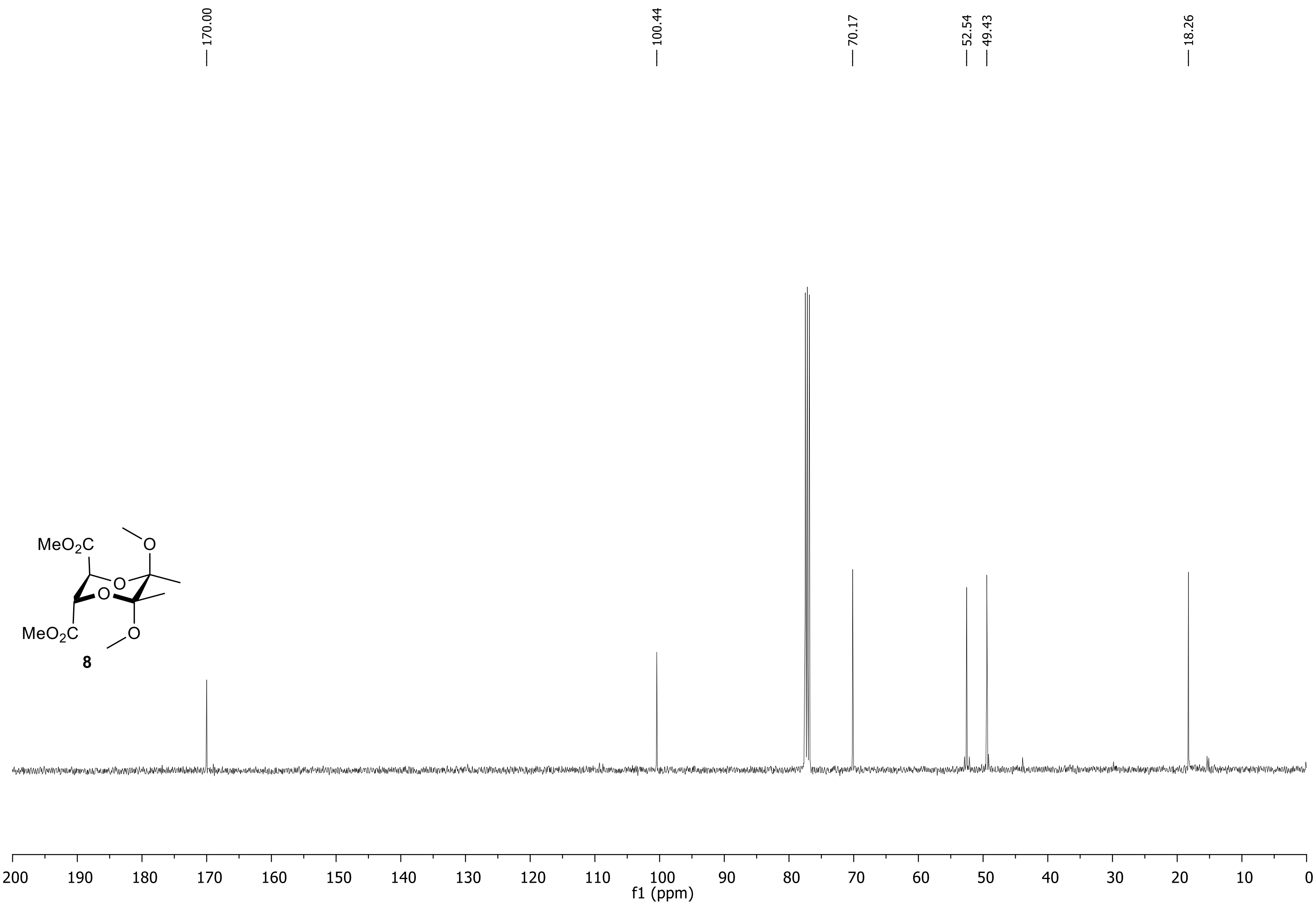

Figure S19 ^{13}C NMR spectrum of (2*S*,3*S*,5*R*,6*R*)-5,6-dimethoxy-5,6-dimethyl-1,4-dioxane-2,3-dimethyl dicarboxylate **8**.

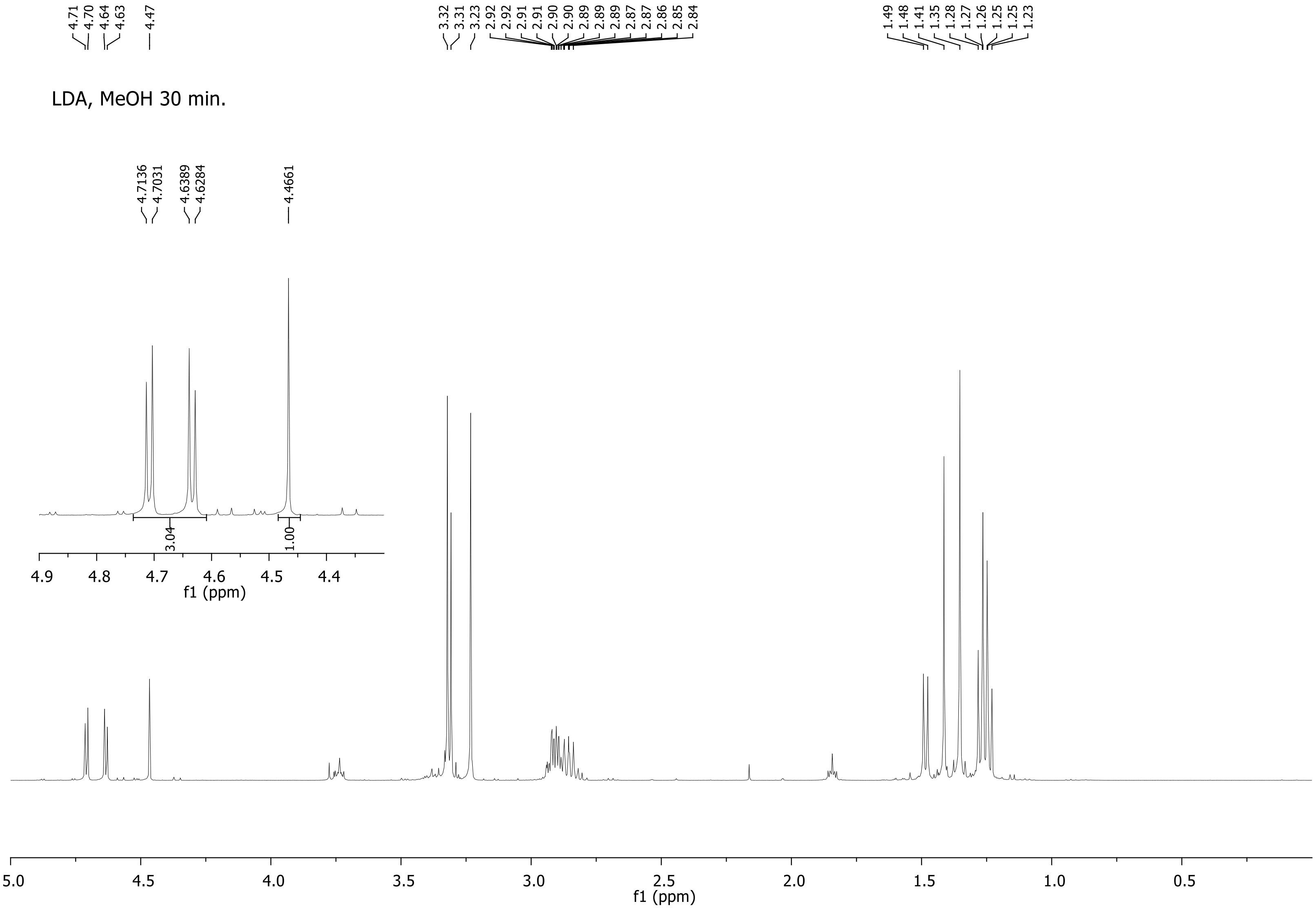

Figure S 20 ^{1}H NMR spectrum of reaction mixture of S2,S3-diethyl (2*R*,3*R*,5*R*,6*R*)-5,6-dimethoxy-5,6-dimethyl-1,4-dioxane-2,3-dicarbothioate **2** with 2.2 eq. of LDA, MeOH, 30 min (Manuscript: Table 1, entry 1).

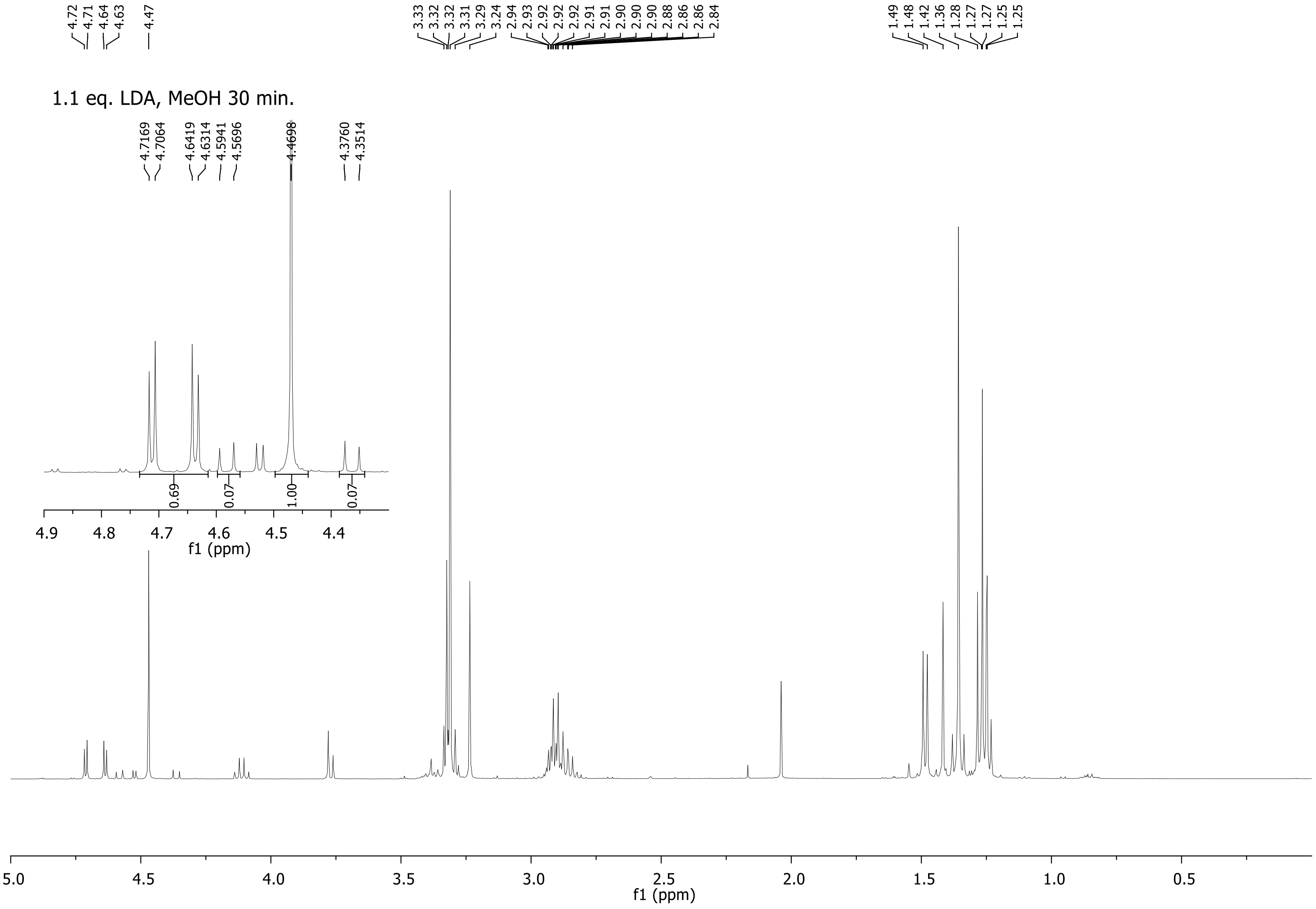

Figure S21 ^{1}H NMR spectrum of reaction mixture of S2,S3-diethyl (2*R*,3*R*,5*R*,6*R*)-5,6-dimethoxy-5,6-dimethyl-1,4-dioxane-2,3-dicarbothioate **2** with 1.1 eq. of LDA, MeOH 30 min (Manuscript: Table 1, entry 2).

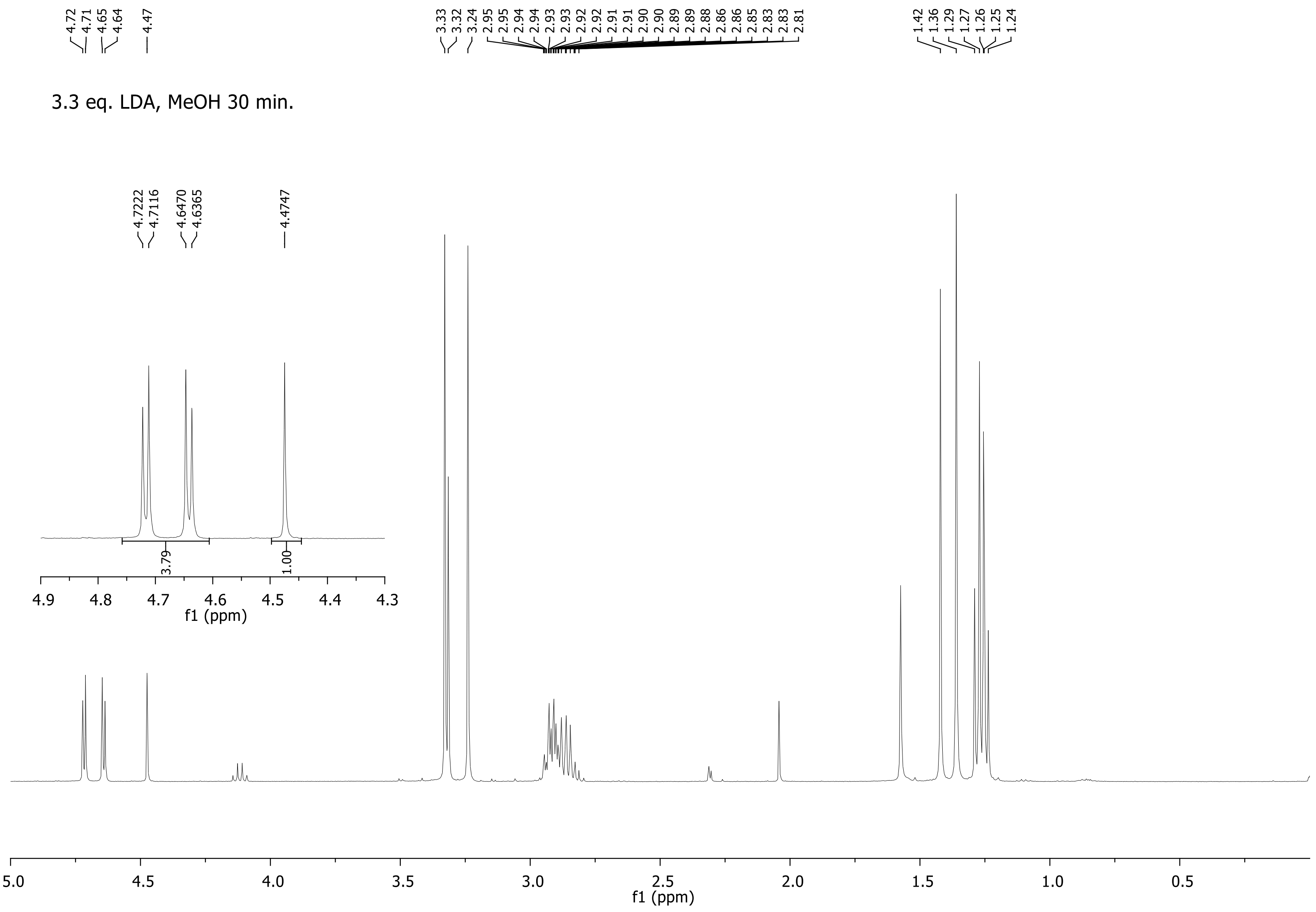

Figure S 22 ^{1}H NMR spectrum of reaction mixture of S2,S3-diethyl (2*R*,3*R*,5*R*,6*R*)-5,6-dimethoxy-5,6-dimethyl-1,4-dioxane-2,3-dicarbothioate **2** with 3.3 eq. of LDA, MeOH 30 min(Manuscript: Table 1, entry 3).

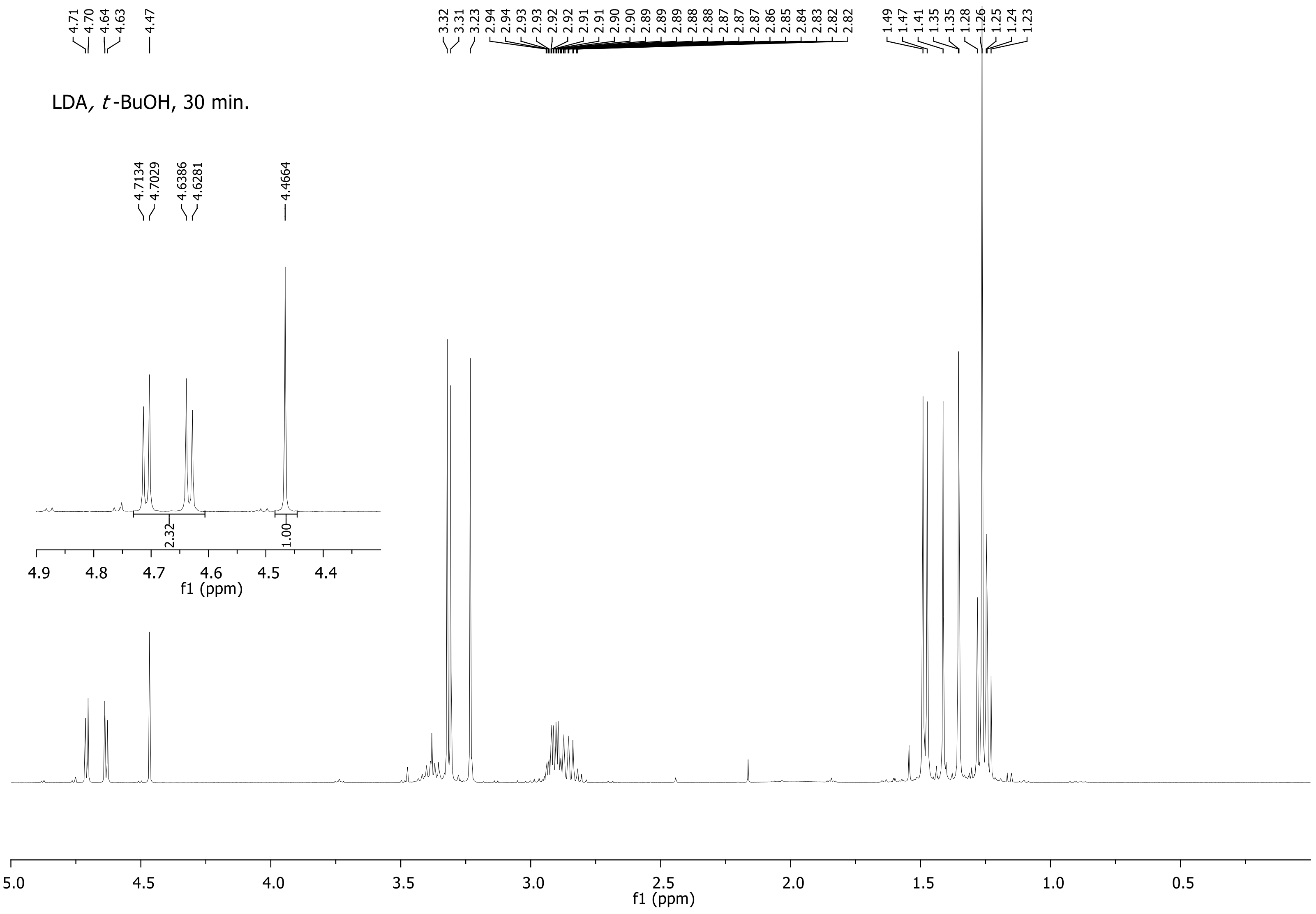

Figure S 23 ^{1}H NMR spectrum of reaction mixture of S2,S3-diethyl (2*R*,3*R*,5*R*,6*R*)-5,6-dimethoxy-5,6-dimethyl-1,4-dioxane-2,3-dicarbothioate **2** with 2.2 eq. of LDA, t-BuOH, 30 min(Manuscript: Table 1, entry 4).

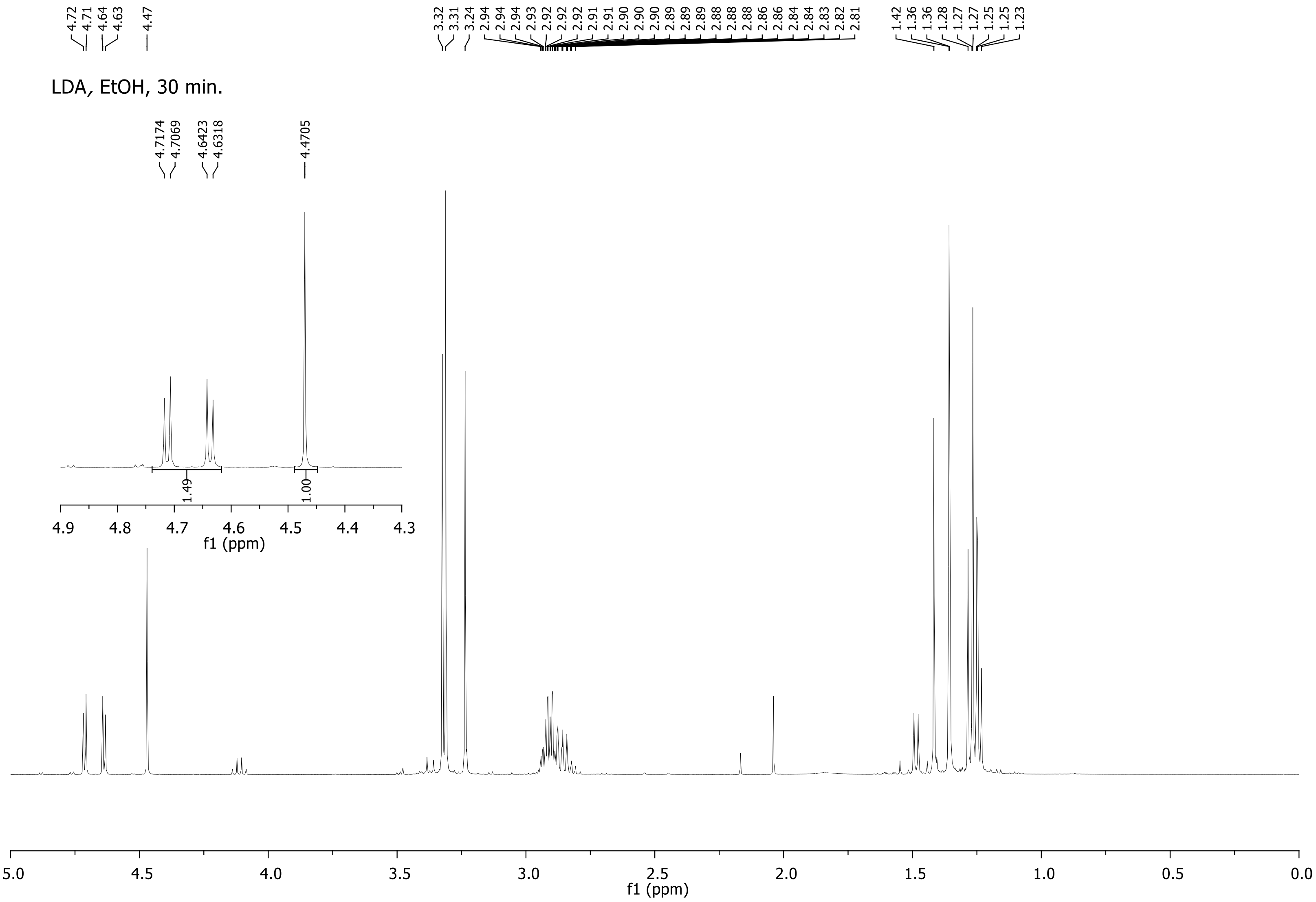

Figure S24 ^{1}H NMR spectrum of reaction mixture of S2,S3-diethyl (2*R*,3*R*,5*R*,6*R*)-5,6-dimethoxy-5,6-dimethyl-1,4-dioxane-2,3-dicarbothioate **2** with 2.2 eq. of LDA, EtOH 30 min (Manuscript: Table 1, entry 5).

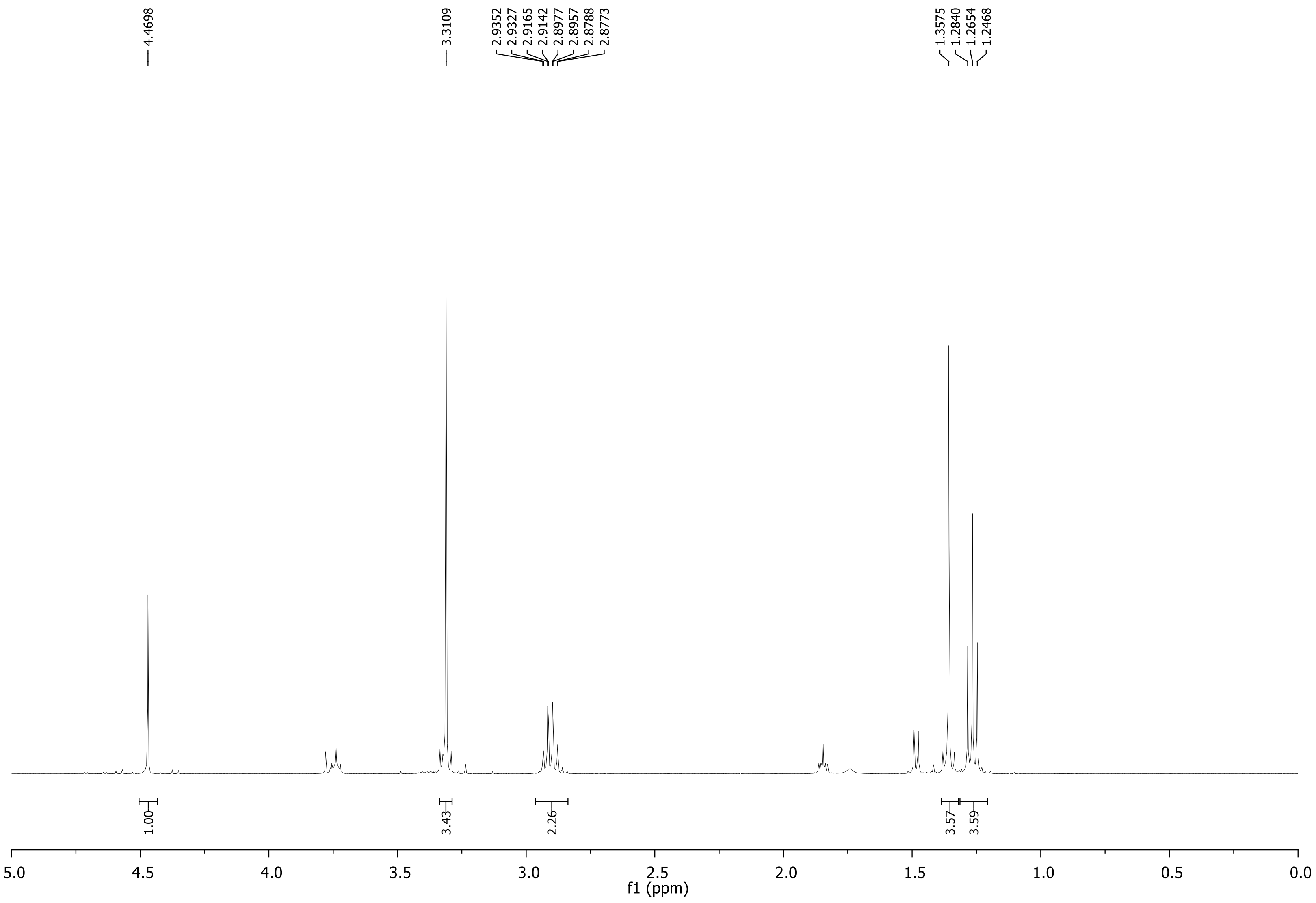

Figure S25 ^{1}H NMR spectrum of reaction mixture of S2,S3-diethyl (2*R*,3*R*,5*R*,6*R*)-5,6-dimethoxy-5,6-dimethyl-1,4-dioxane-2,3-dicarbothioate **2** with 2.2 eq. of LDA, MeOH 0 min (Manuscript: Table 1, entry 6).

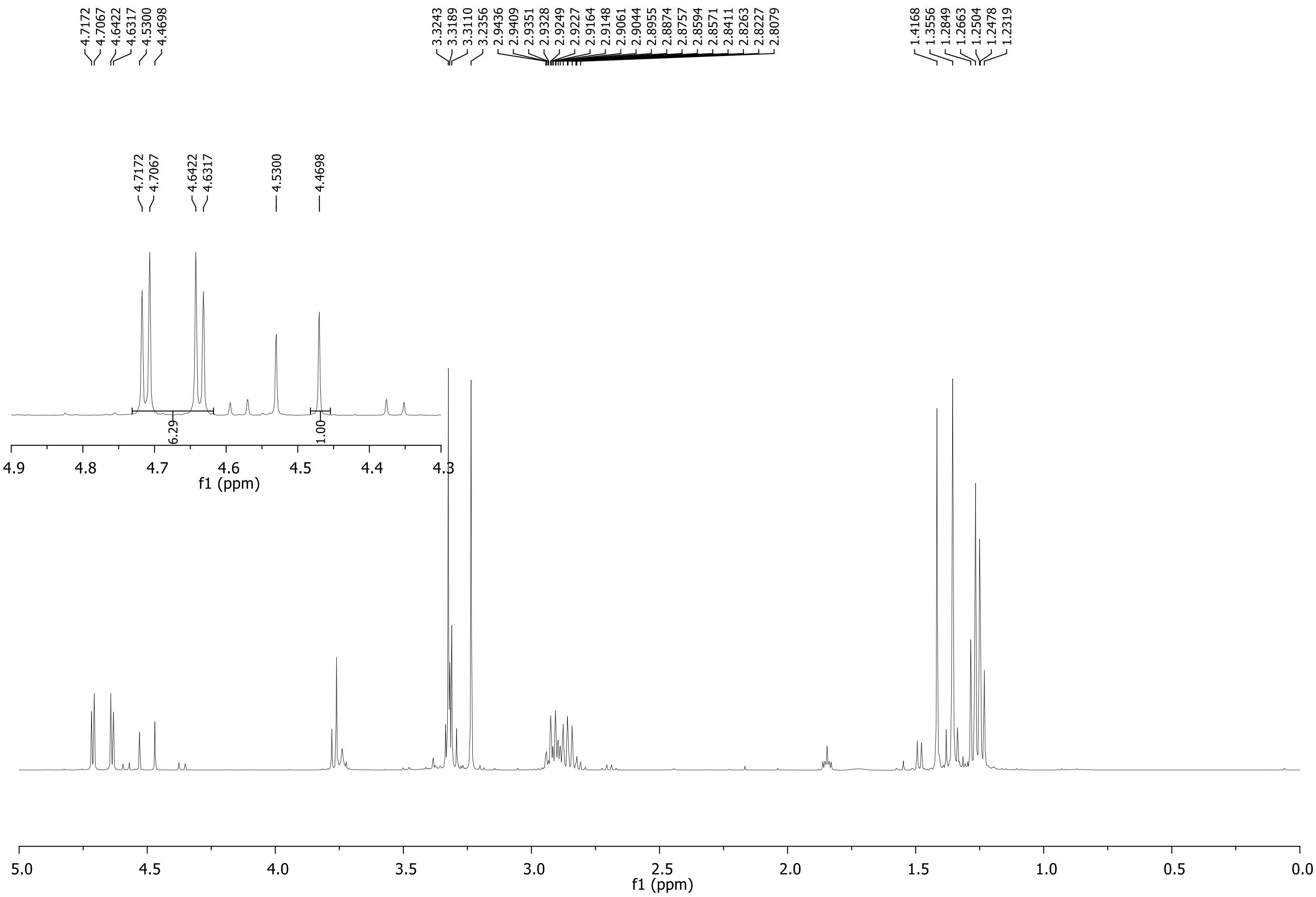

Figure S27 ^{1}H NMR spectrum of reaction mixture of S2,S3-diethyl (2*R*,3*R*,5*R*,6*R*)-5,6-dimethoxy-5,6-dimethyl-1,4-dioxane-2,3-dicarbothioate 2 with 2.2 eq. of LDA, MeOH 15 min (Manuscript: Table 1, entry 7).

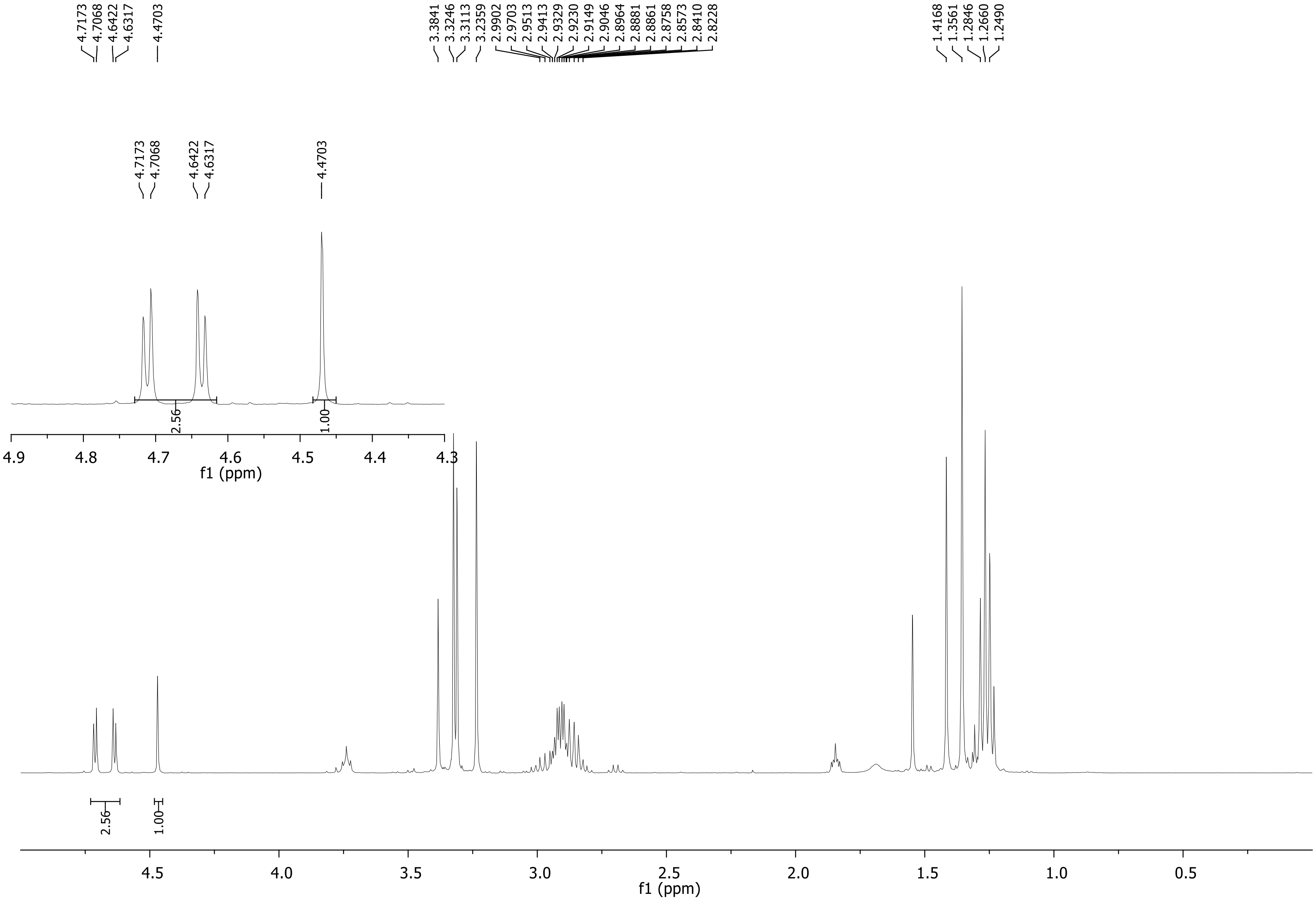

Figure S26 ^{1}H NMR spectrum of reaction mixture of S2,S3-diethyl (2*R*,3*R*,5*R*,6*R*)-5,6-dimethoxy-5,6-dimethyl-1,4-dioxane-2,3-dicarbothioate **2** with 2.2 eq. of LDA, MeOH 120 min (Manuscript: Table 1, entry 8).

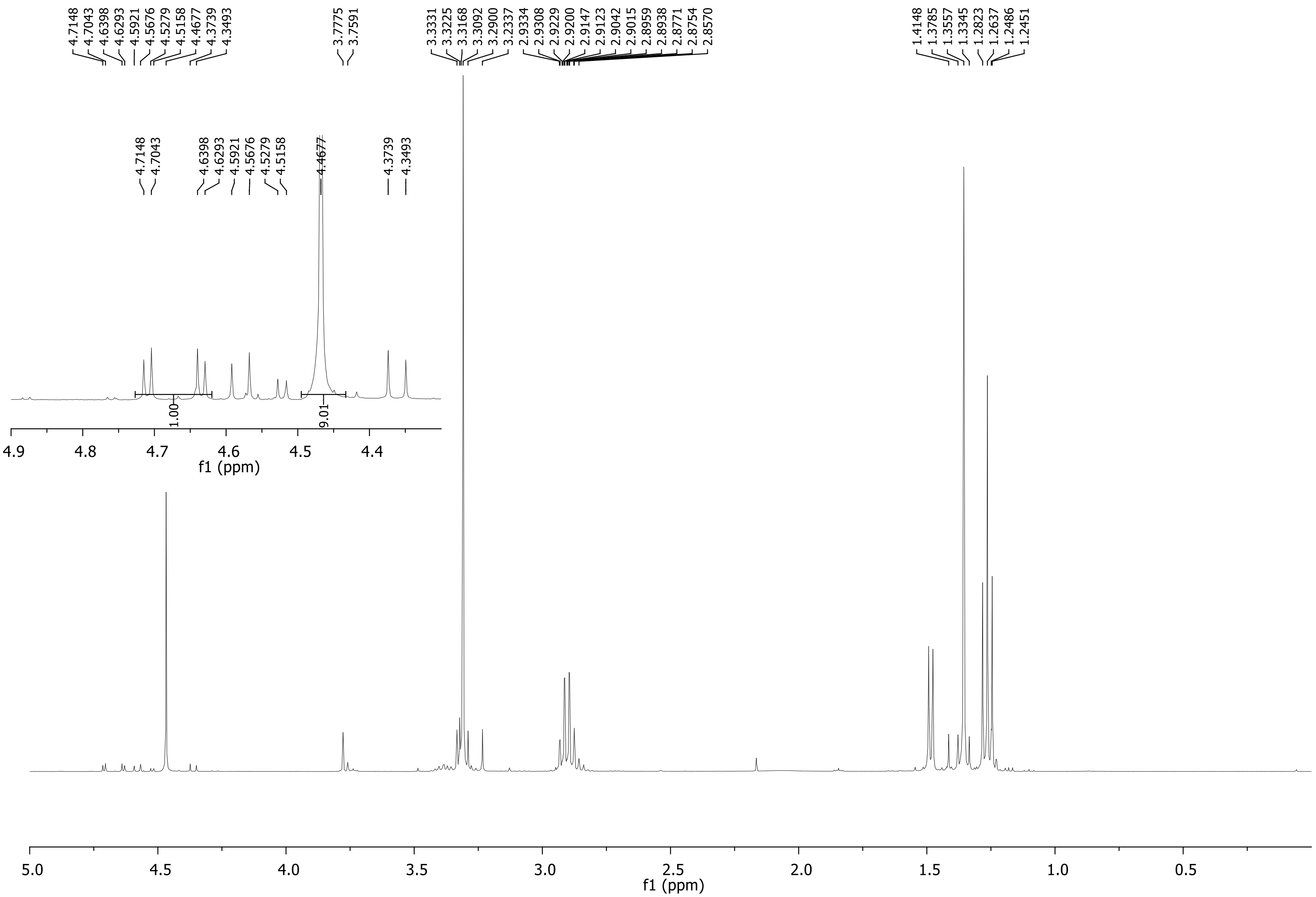

Figure S27 ^{1}H NMR spectrum of reaction mixture of S2,S3-diethyl (2*R*,3*R*,5*R*,6*R*)-5,6-dimethoxy-5,6-dimethyl-1,4-dioxane-2,3-dicarbothioate **2** with 2.2 eq. of LDA, – 100 °C, MeOH, 30 min (Manuscript: Table 1, entry 9).

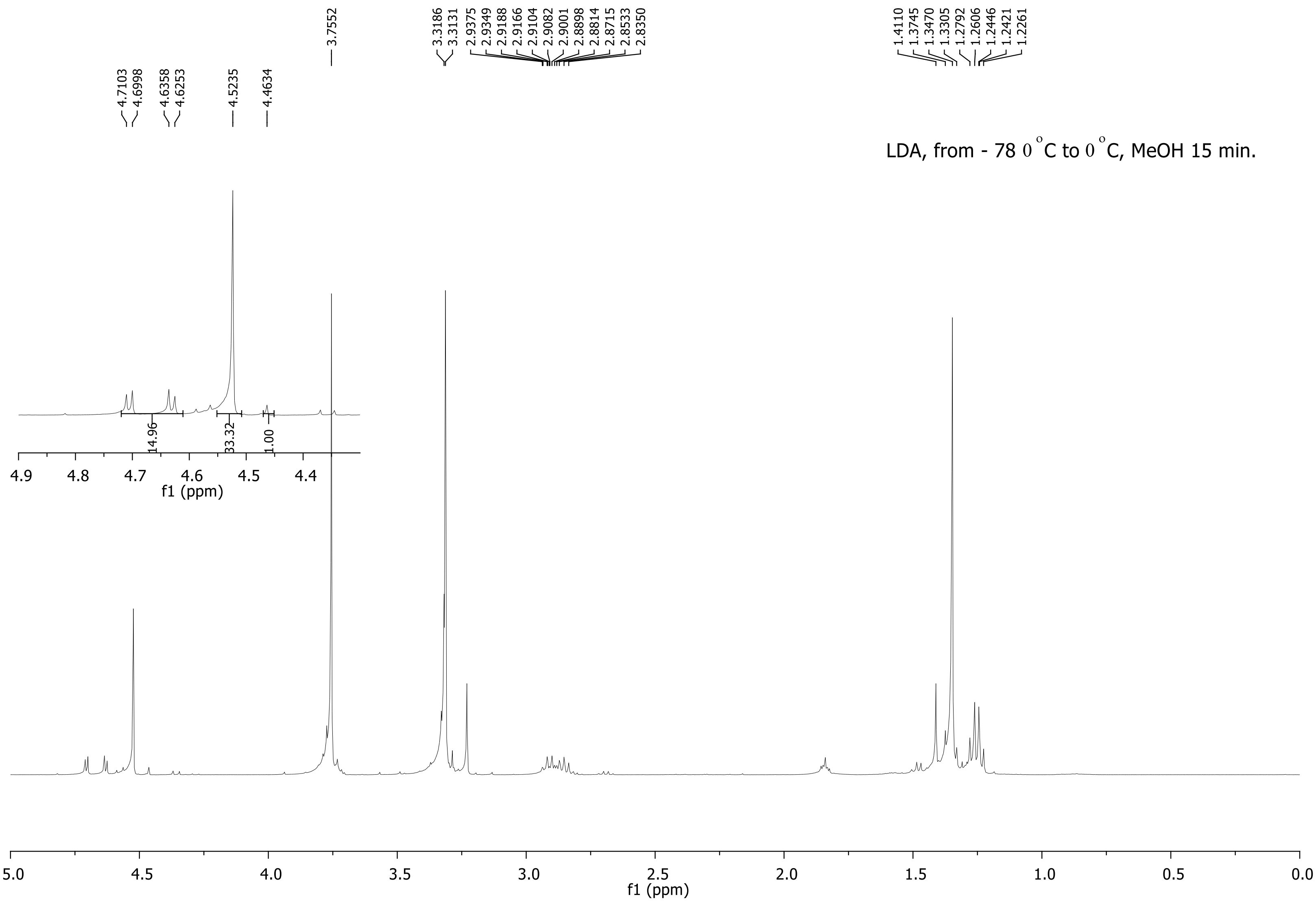

Figure S28 ^{1}H NMR spectrum of reaction mixture of S2,S3-diethyl (2*R*,3*R*,5*R*,6*R*)-5,6-dimethoxy-5,6-dimethyl-1,4-dioxane-2,3-dicarbothioate **2** with 2.2 eq. of LDA, MeOH from – 78 °C to 0 °C.,30 min (Manuscript: Table 1, entry 10).

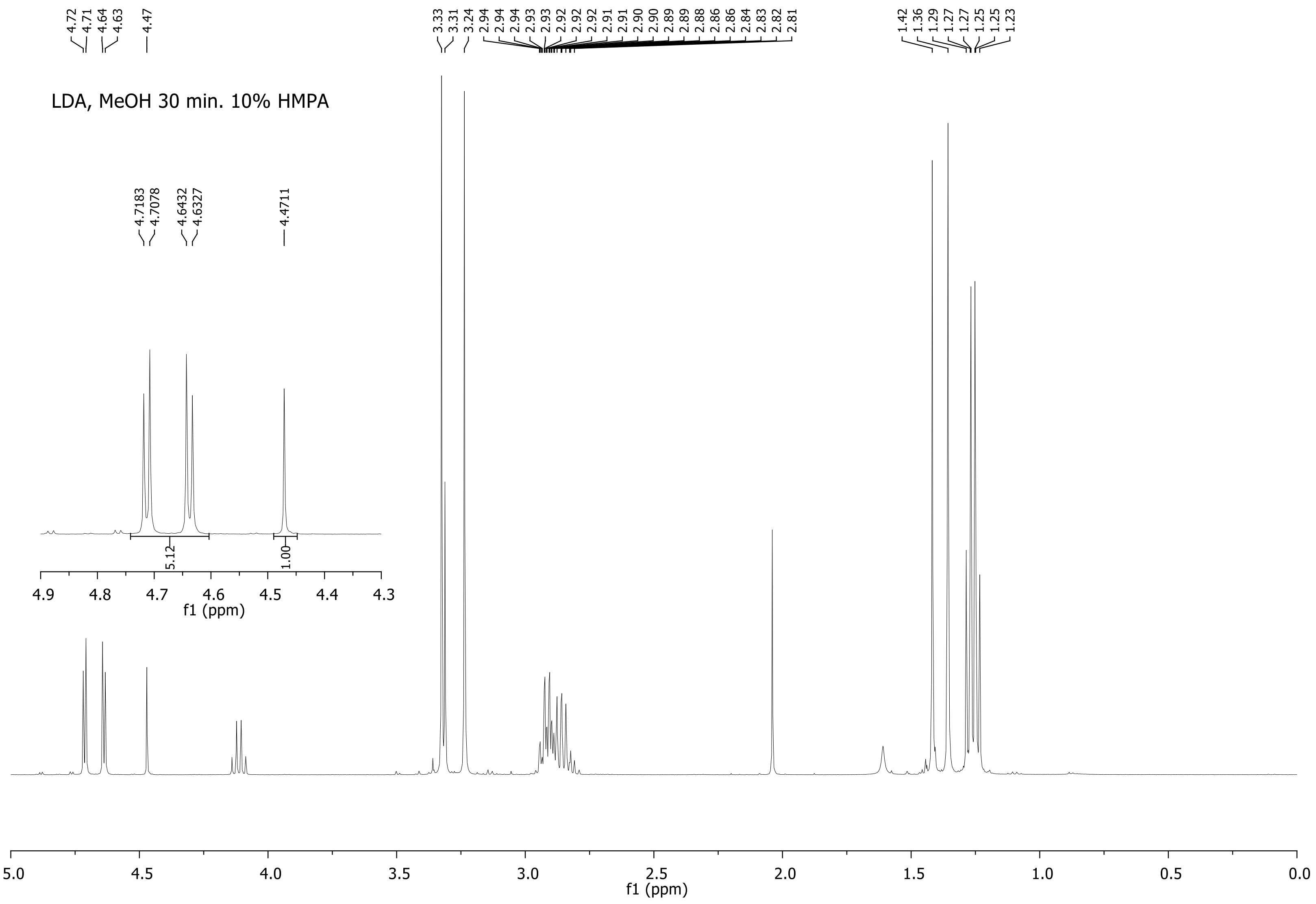

Figure S29 ^{1}H NMR spectrum of reaction mixture of S2,S3-diethyl (2*R*,3*R*,5*R*,6*R*)-5,6-dimethoxy-5,6-dimethyl-1,4-dioxane-2,3-dicarbothioate **2** with 2.2 eq. of LDA, MeOH, 10% HMPA, 30 min (Manuscript: Table 1, entry 11**).**

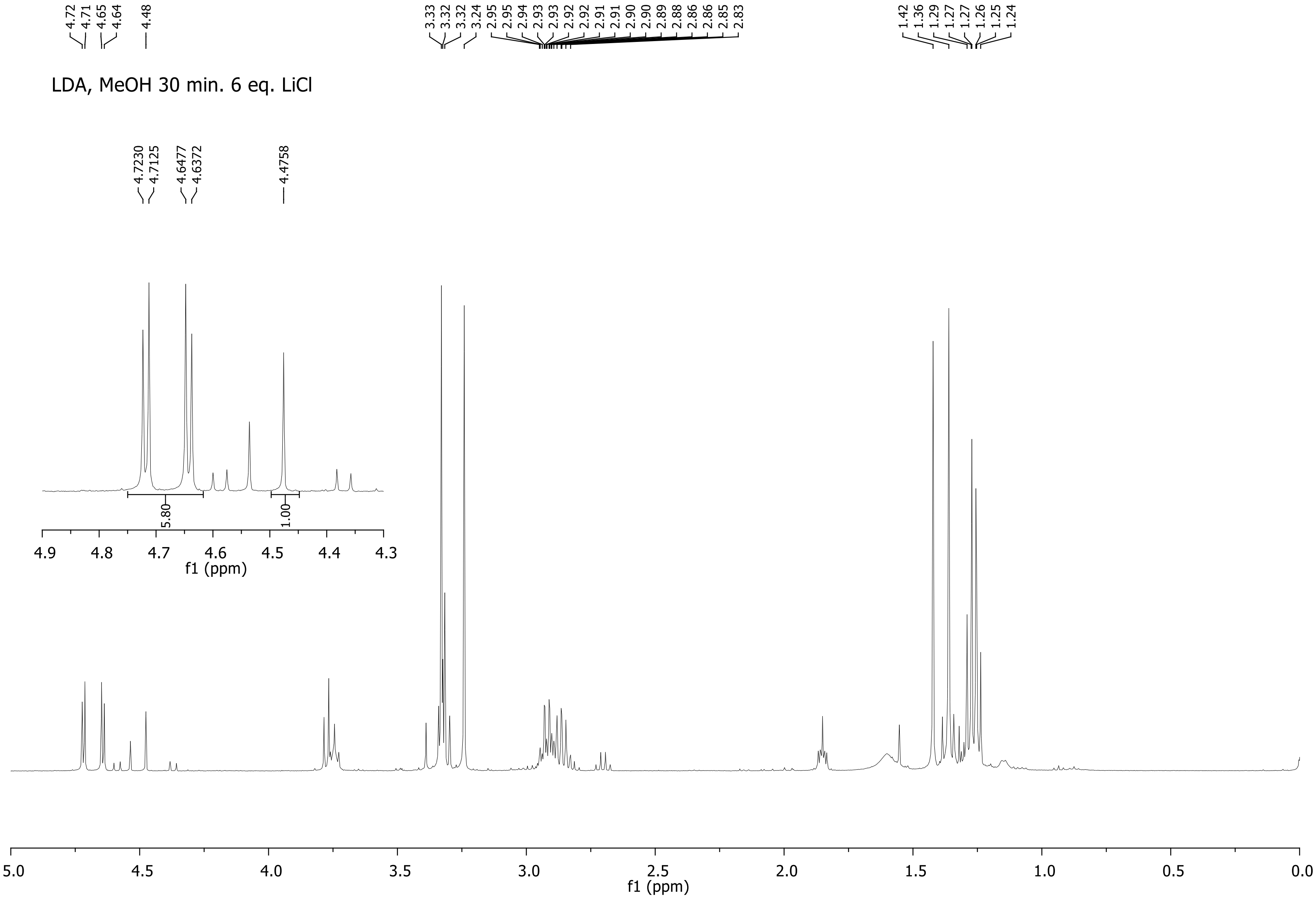

Figure S30 ^{1}H NMR spectrum of reaction mixture of S2,S3-diethyl (2*R*,3*R*,5*R*,6*R*)-5,6-dimethoxy-5,6-dimethyl-1,4-dioxane-2,3-dicarbothioate **2** with 2.2 eq. of LDA, MeOH, 6 eq. LiCl, 30 min (Manuscript: Table 1, entry 12).

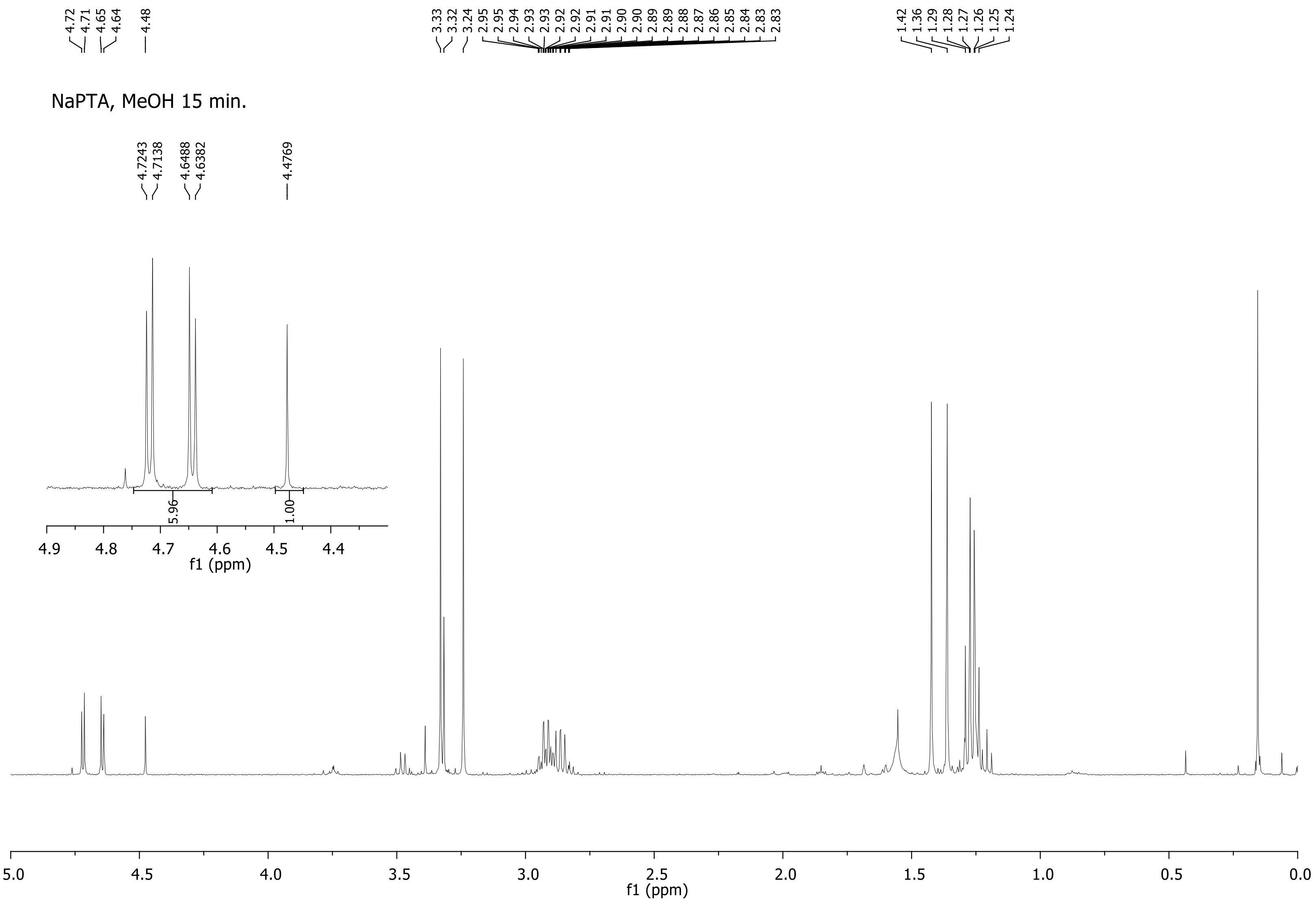

Figure S31 ^{1}H NMR spectrum of reaction mixture of S2,S3-diethyl (2*R*,3*R*,5*R*,6*R*)-5,6-dimethoxy-5,6-dimethyl-1,4-dioxane-2,3-dicarbothioate **2** with 2.2 eq. of NaPTA, MeOH 30 min (Manuscript: Table 1, entry 13).

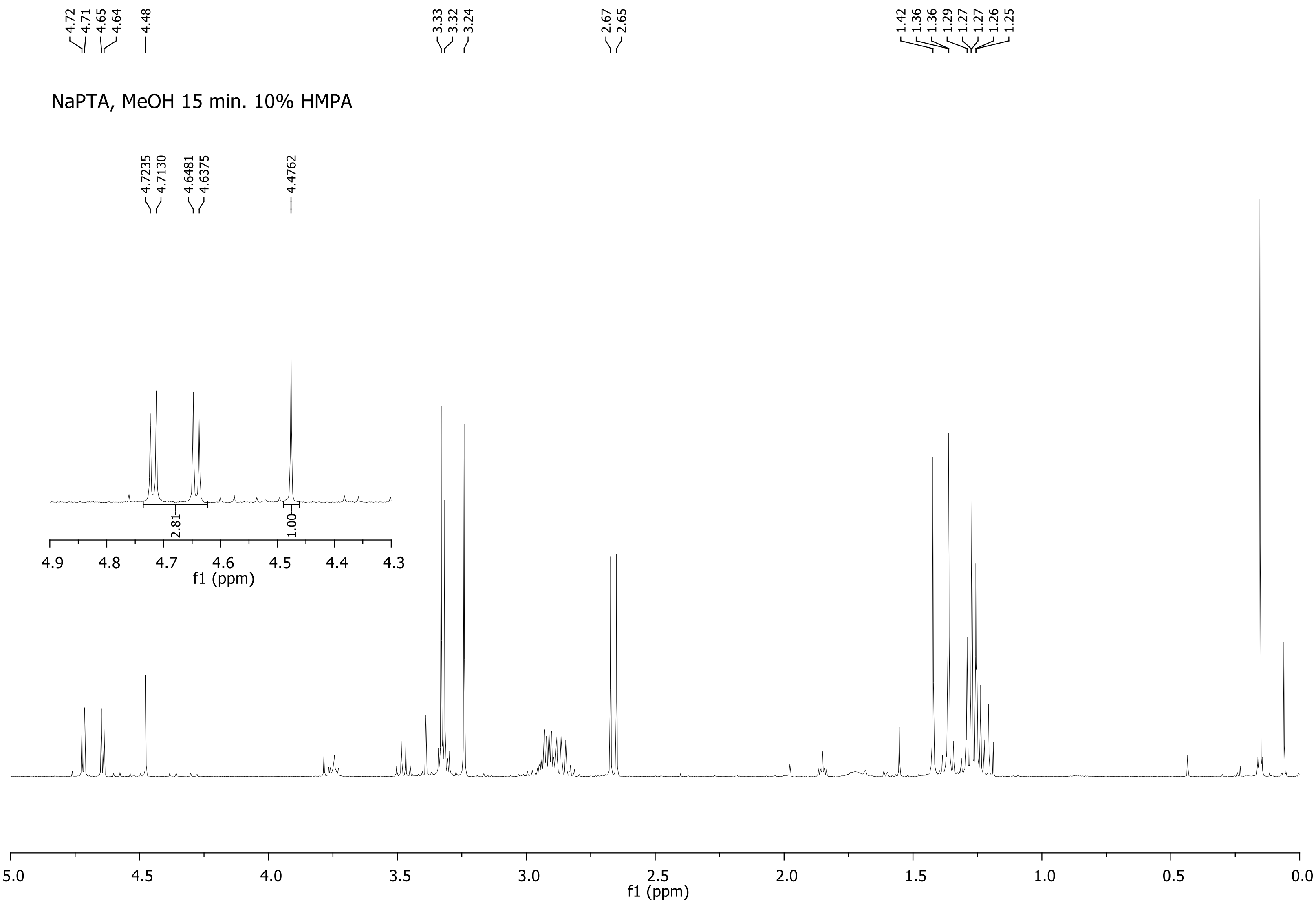

Figure S32 ^{1}H NMR spectrum of reaction mixture of S2,S3-diethyl (2*R*,3*R*,5*R*,6*R*)-5,6-dimethoxy-5,6-dimethyl-1,4-dioxane-2,3-dicarbothioate **2** with 2.2 eq. of NaPTA, MeOH, 10% HMPA, 30 min (Manuscript: Table 1, entry 14).

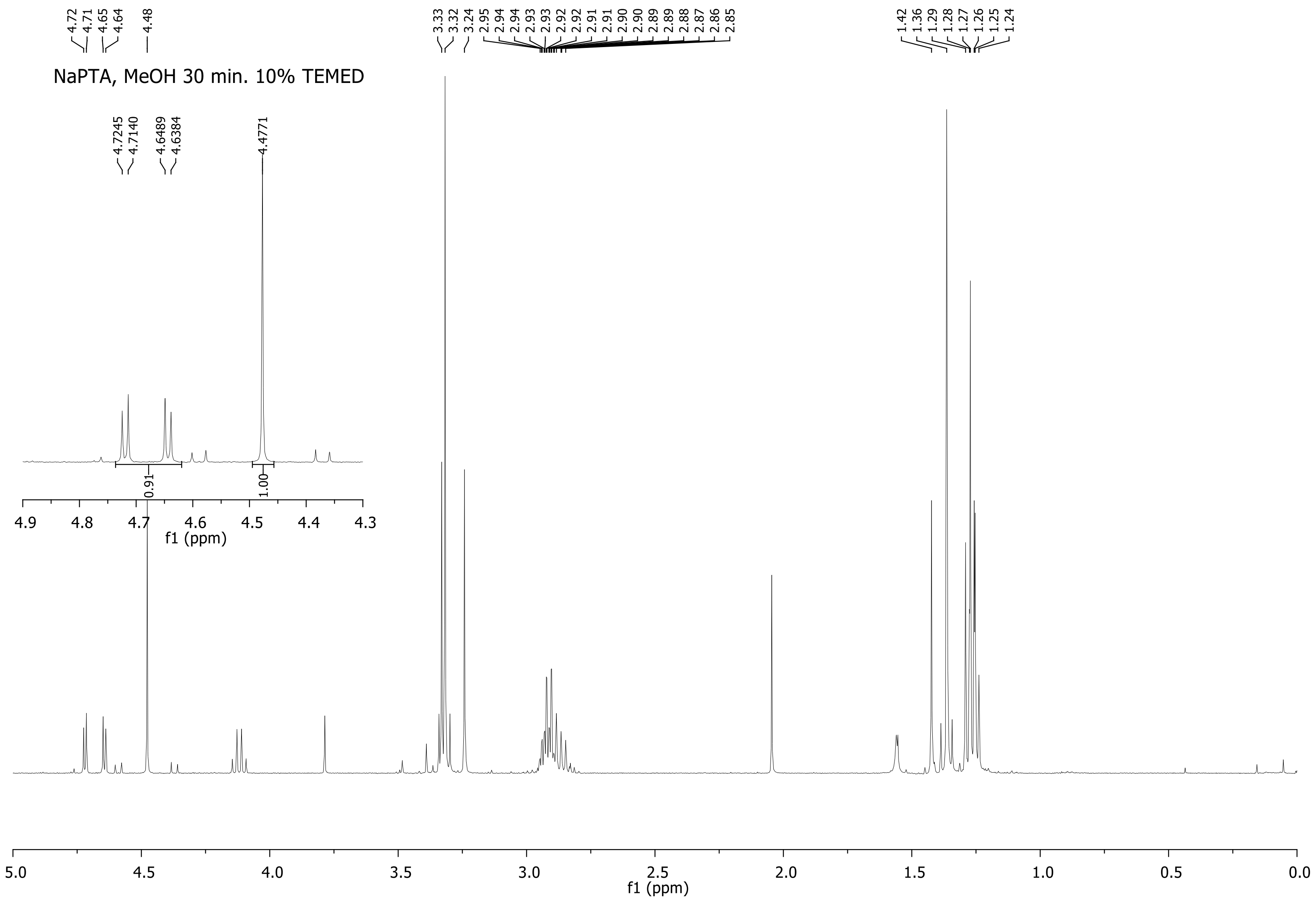

Figure S33 ^{1}H NMR spectrum of reaction mixture of S2,S3-diethyl (2*R*,3*R*,5*R*,6*R*)-5,6-dimethoxy-5,6-dimethyl-1,4-dioxane-2,3-dicarbothioate **2** with 2.2 eq. of NaPTA, MeOH,, 10% TEMED, 30 min(Manuscript: Table 1, entry 15).

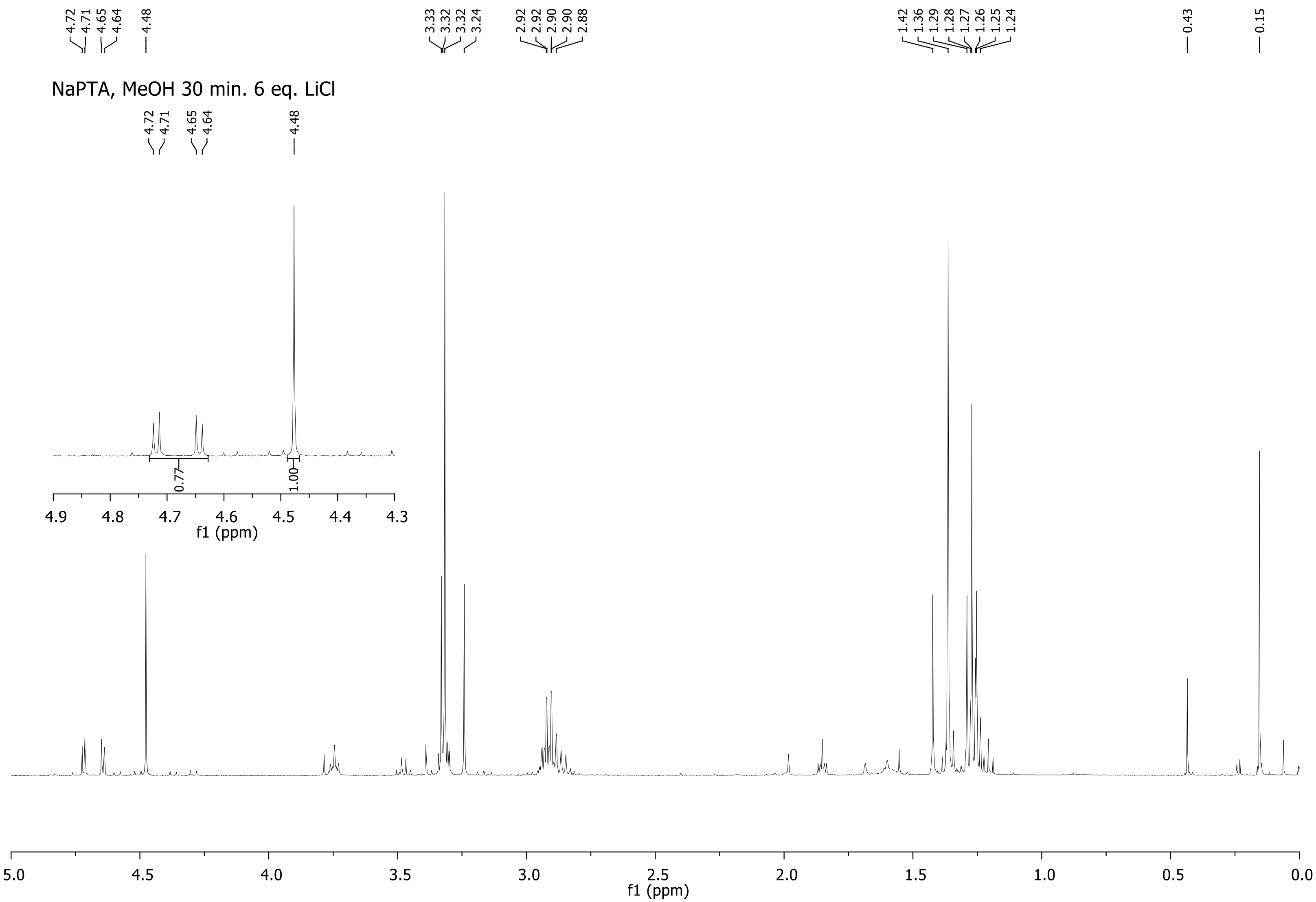

Figure S34 ^{1}H NMR spectrum of reaction mixture of S2,S3-diethyl (2*R*,3*R*,5*R*,6*R*)-5,6-dimethoxy-5,6-dimethyl-1,4-dioxane-2,3-dicarbothioate **2** with 2.2 eq. of NaPTA, MeOH, 6 eq. LiCl 30 min (Manuscript: Table 1, entry 16).

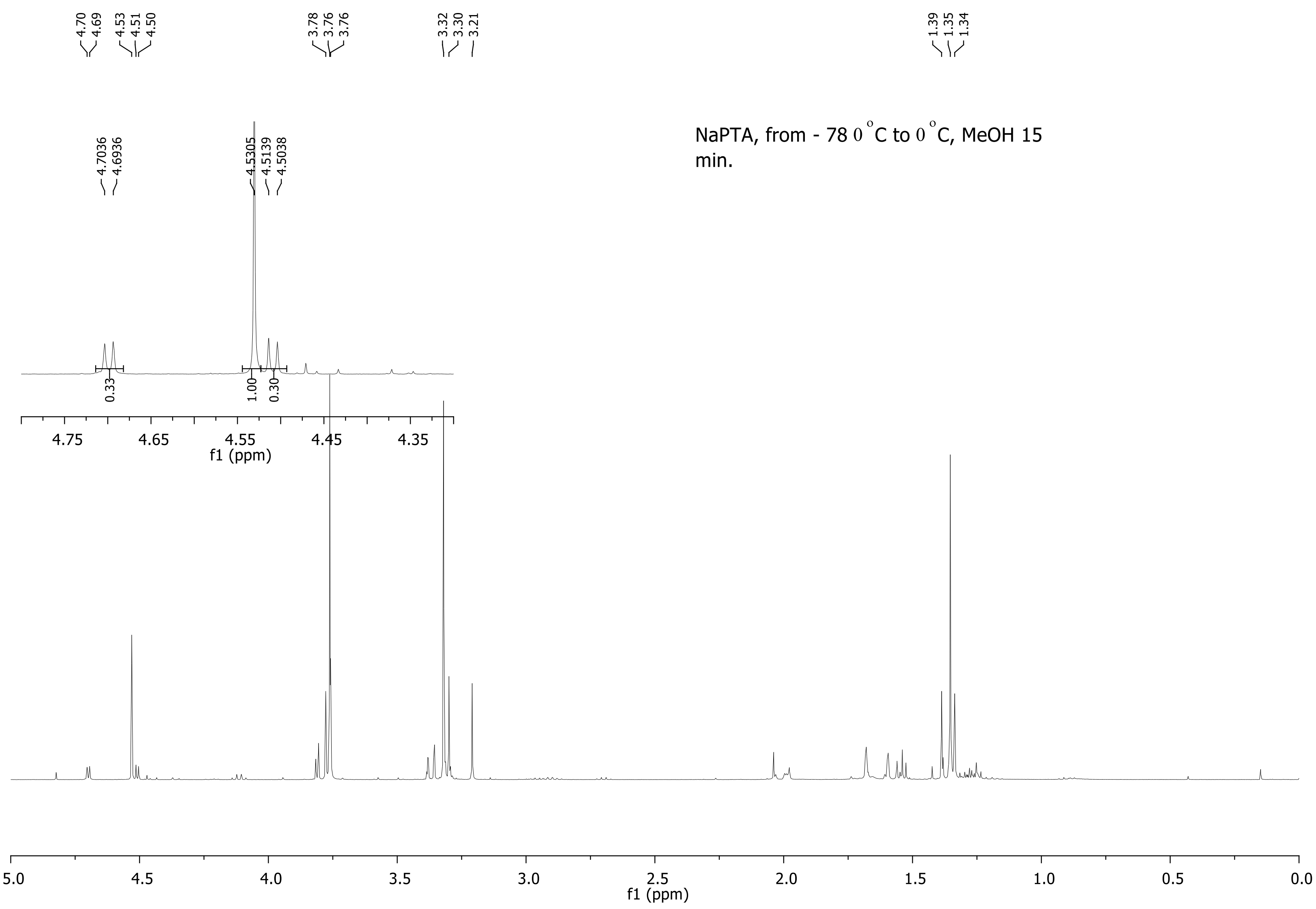

Figure S37 ^{1}H NMR spectrum of reaction mixture of S2,S3-diethyl (2*R*,3*R*,5*R*,6*R*)-5,6-dimethoxy-5,6-dimethyl-1,4-dioxane-2,3-dicarbothioate **2** with 2.2 eq. of NaPTA, MeOH, from – 78 °C to 0 °C., 30 min (Manuscript: Table 1, entry 17).

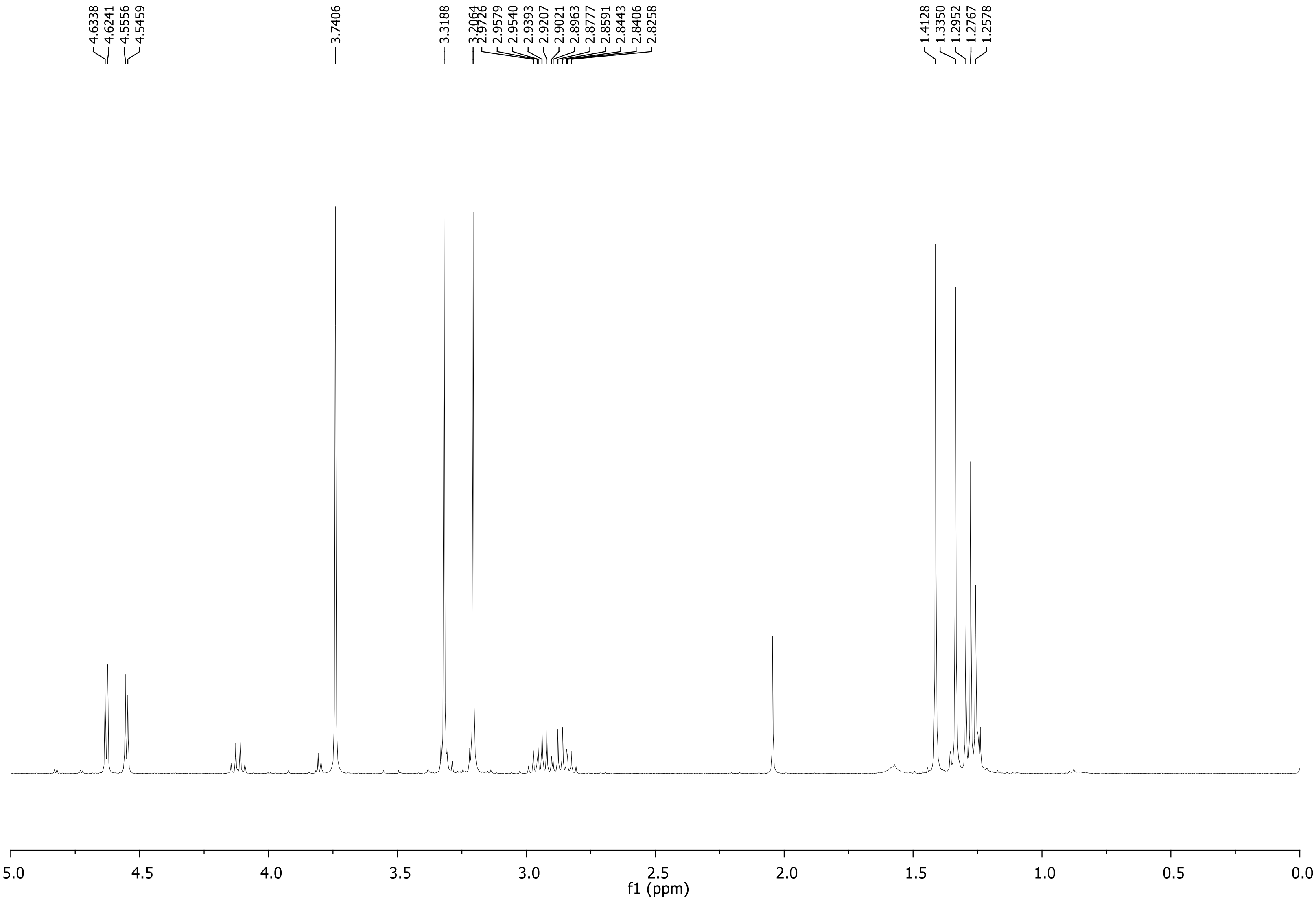

Figure S35 ^{1}H NMR spectrum of reaction mixture of methyl (2*R*,3*R*,5R,6*R*)-3-ethylsulfanylcarbonyl-5,6-dimethoxy-5,6-dimethyl-1,4-dioxane-2-carboxylate **3** with 2.2 eq. of NaPTA, MeOH 30 min.

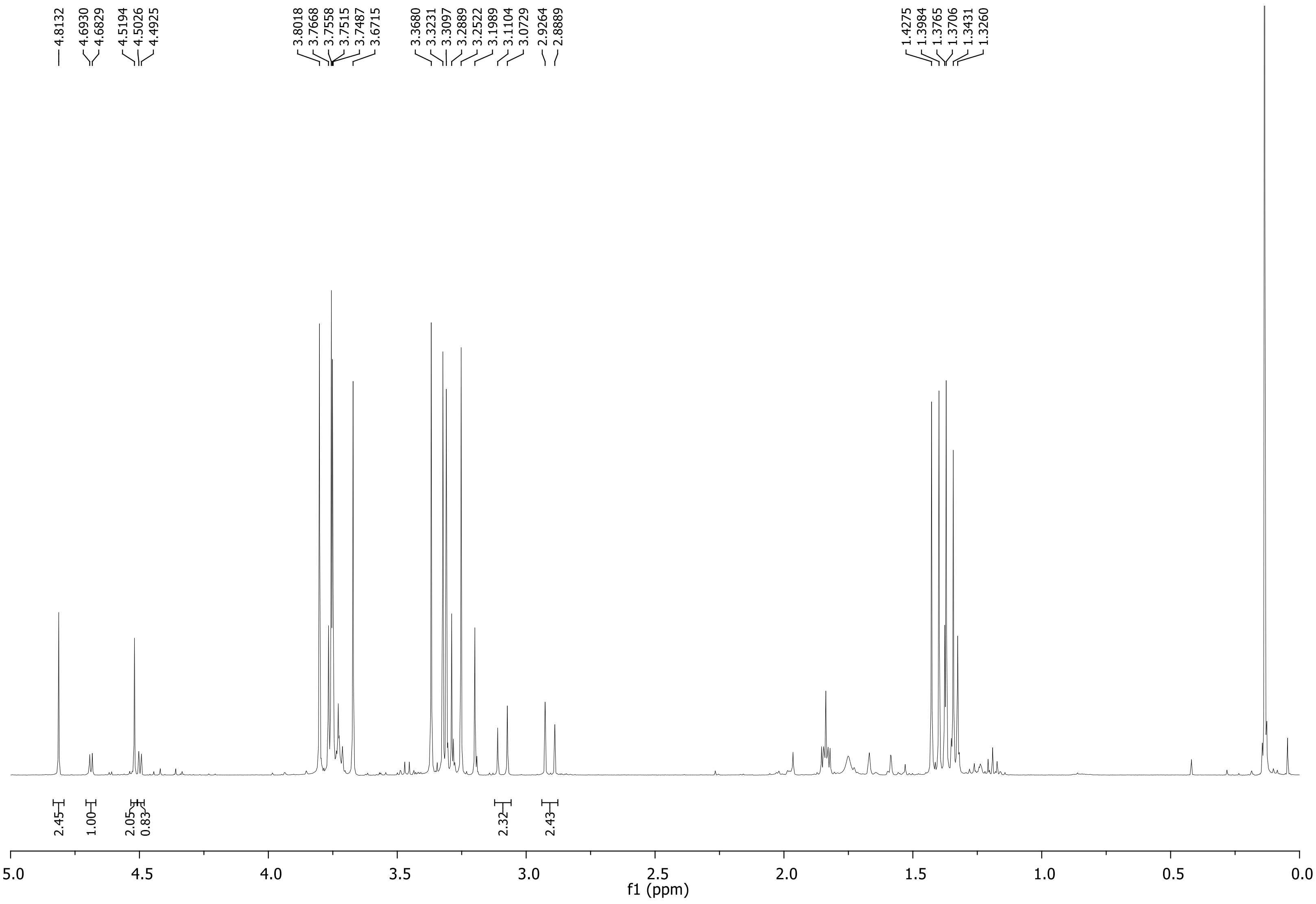

Figure S36 ^{1}H NMR spectrum of crude reaction mixture of (2*R*,3*R*,5*R*,6*R*)-5,6-dimethoxy-5,6-dimethyl-1,4-dioxane-2,3-dimethyl dicarboxylate **1** with NaPTA, MeOH, - 78 ℃ to 0 ℃, 30 min.
